# Expression of *P. falciparum var* Genes Involves Exchange of the Histone Variant H2A.Z at the Promoter

**DOI:** 10.1371/journal.ppat.1001292

**Published:** 2011-02-17

**Authors:** Michaela Petter, Chin Chin Lee, Timothy J. Byrne, Katja E. Boysen, Jennifer Volz, Stuart A. Ralph, Alan F. Cowman, Graham V. Brown, Michael F. Duffy

**Affiliations:** 1 Department of Medicine, Royal Melbourne Hospital, University of Melbourne, Melbourne, Australia; 2 The Walter and Eliza Hall Institute of Medical Research, Melbourne, Australia; 3 Department of Biochemistry and Molecular Biology, Bio21 Molecular Sciences and Biotechnology Institute, University of Melbourne, Melbourne, Australia; 4 Department of Medical Biology, University of Melbourne, Melbourne, Australia; Weill Medical College of Cornell University, United States of America

## Abstract

*Plasmodium falciparum* employs antigenic variation to evade the human immune response by switching the expression of different variant surface antigens encoded by the *var* gene family. Epigenetic mechanisms including histone modifications and sub-nuclear compartmentalization contribute to transcriptional regulation in the malaria parasite, in particular to control antigenic variation. Another mechanism of epigenetic control is the exchange of canonical histones with alternative variants to generate functionally specialized chromatin domains. Here we demonstrate that the alternative histone PfH2A.Z is associated with the epigenetic regulation of *var* genes. In many eukaryotic organisms the histone variant H2A.Z mediates an open chromatin structure at promoters and facilitates diverse levels of regulation, including transcriptional activation. Throughout the asexual, intraerythrocytic lifecycle of *P. falciparum* we found that the *P. falciparum* ortholog of H2A.Z (PfH2A.Z) colocalizes with histone modifications that are characteristic of transcriptionally-permissive euchromatin, but not with markers of heterochromatin. Consistent with this finding, antibodies to PfH2A.Z co-precipitate the permissive modification H3K4me3. By chromatin-immunoprecipitation we show that PfH2A.Z is enriched in nucleosomes around the transcription start site (TSS) in both transcriptionally active and silent stage-specific genes. In *var* genes, however, PfH2A.Z is enriched at the TSS only during active transcription in ring stage parasites. Thus, in contrast to other genes, temporal *var* gene regulation involves histone variant exchange at promoter nucleosomes. Sir2 histone deacetylases are important for *var* gene silencing and their yeast ortholog antagonises H2A.Z function in subtelomeric yeast genes. In immature *P. falciparum* parasites lacking Sir2A or Sir2B high *var* transcription levels correlate with enrichment of PfH2A.Z at the TSS. As Sir2A knock out parasites mature the *var* genes are silenced, but PfH2A.Z remains enriched at the TSS of *var* genes; in contrast, PfH2A.Z is lost from the TSS of de-repressed *var* genes in mature Sir2B knock out parasites. This result indicates that PfH2A.Z occupancy at the active *var* promoter is antagonized by PfSir2A during the intraerythrocytic life cycle. We conclude that PfH2A.Z contributes to the nucleosome architecture at promoters and is regulated dynamically in active *var* genes.

## Author Summary

*Plasmodium falciparum* is a protist parasite that causes malaria and kills more than 800,000 people per year. The parasite escapes from the human immune response by antigenic variation through switching between expression of different *var* genes. These encode different variant antigens that are expressed on the surface of the infected erythrocyte and mediate pathogenic adhesion of the infected erythrocytes to host receptors. Understanding how this process is regulated may lead to the identification of factors that are essential for immune evasion and that could represent novel drug targets. Here, we have identified the parasite's histone variant PfH2A.Z as a novel contributor to the transcriptional regulation of antigenic variation. PfH2A.Z is enriched in the promoter of many genes, but enrichment correlates with gene expression only in *var* genes. Furthermore we show that PfH2A.Z enrichment in *var* promoters is antagonised by the *var* gene silencing factor PfSir2A. These findings further extend our knowledge of the complex mechanisms regulating gene expression in *P. falciparum.*

## Introduction

*Plasmodium falciparum* causes the majority of malaria-induced morbidity and mortality, resulting in approximately 860,000 deaths each year [1]. *Plasmodium* parasites have a complicated life cycle, during which they differentiate into several morphologically distinct asexual and sexual stages in the human host and the mosquito vector.

Disease occurs during the repeated cycles of invasion and asexual replication of the parasite inside human erythrocytes. A central mechanism of malaria pathogenesis is the ability of the infected erythrocytes (IE) to sequester at vascular sites by cytoadherence to host receptors. Through this process the parasite avoids clearance by splenic macrophages and contributes to severe malaria complications such as cerebral and placental malaria [2]. Sequestration is mediated by the *P. falciparum* erythrocyte membrane protein 1 (PfEMP1) variant antigens, which are expressed on the IE surface and are encoded by approximately 60 genes of the *var* multigene family [3], [4], [5], [6]. Only a single *var* gene is expressed at a time [7], [8] and switching between expression of different PfEMP1 variants alters both the cytoadherence phenotype and the antigenic profile of the IE, resulting in antigenic variation [9]. These processes are critical to immune evasion of *P. falciparum* and chronic infection [10], [11], [12].

During its asexual, intaerythrocytic life cycle, *P. falciparum* employs stringent regulatory mechanisms to achieve stage-specific gene expression [13], [14]. The multiple layers of regulation that mediate morphological and physiological adaptations, include specific transcription factors and repressors [15], [16], [17], [18], translational repression [19], [20], post-translational protein modifications (reviewed [21]) and epigenetic mechanisms [22], [23]. Recent studies assessing the global structure of chromatin during the asexual intra-erythrocytic developmental cycle (IDC) have shown that chromatin undergoes massive reorganization in *P. falciparum*, emphasizing the significance of epigenetic control in the parasite [24].

Epigenetic gene regulation confers a heritable state of gene expression and is typically mediated by changes in chromatin structure without a change in DNA sequence. The fundamental units of chromatin are nucleosomes and are formed by ∼146 bp of DNA wrapping around an octamer of histones. The canonical nucleosome components are two H2A/H2B dimers and an H3/H4 tetramer. In most eukaruyotes an additional subunit, H1, links nucleosomes, but this subunit is missing in Plasmodium species. Chromatin can exist as compact, silent heterochromatin and as open, transcriptionally competent euchromatin. These different physical states are essentially determined by the composition and distribution of nucleosomes, post-translational modification of histones, presence of chromatin associated trans-factors and by the covalent modification of DNA, although this latter mechanism has not yet been shown in *Plasmodium*. The *Plasmodium* genome encodes a broad set of common chromatin remodelling and modifying factors, including many that are novel and that may be employed in unique epigenetic mechanisms [25], [26], [27].

The organisation of *P. falciparum* chromatin has unique features that differ markedly from other eukaryotes. *P. falciparum* intergenic regions, including promoters, display a global nucleosome depletion [24], [28]. Consequently, and unlike in other eukaryotes, nucleosome occupancy at *P. falciparum* promoters does not correlate inversely with steady-state mRNA levels [29], [30], [31]. Genome-wide analysis of histone modifications has shown that the blood stage genome of *P. falciparum* exists in an unusually euchromatic state conferred by the euchromatin marks trimethylated lysine 4 of histone 3 (H3K4me3) and acetylated lysine 9 of histone 3 (H3K9ac) [22], [32]. In contrast to the situation in yeast and other eukaryotes, the presence of neither H3K4me3 nor H3K9ac enrichment seems to correlate with gene activity throughout most of the intraerythrocytic cycle, except for late stage schizont parasites when these marks are enriched at the 5′ ends of active genes [22]. *P. falciparum* heterochromatin, defined by the histone post-translational modification H3K9me3 and its cognate trans-factor heterochromatin protein 1 (HP1), is restricted to subtelomeric and several chromosome internal domains in *P. falciparum* that contain gene families including the majority of *var* genes [22], [33], [34], [35], [36].

The *var* genes present both in subtelomeric and central chromosomal positions form clusters at the nuclear periphery [37], [38], [39] and expression of a *var* gene appears to require it to leave the heterochromatic cluster and relocate to a specific perinuclear expression site [35], [38], [40], [41]. The variegated, monoallelic expression of *var* genes is controlled by epigenetic mechanisms [42]. To become activated in ring stages, a *var* gene must acquire the histone marks H3K4me3 and H3K9ac in its promoter [34], [35]. The *var* promoter is the only *cis* element required for monoallelic exclusive transcription [8] but promoter activity of the *var* gene intron is important for *var* gene silencing [43], [44], [45], [46] and additional *cis* sequence elements contribute to the rate of *var* gene switching [47]. A critical role of histone acetylation in *var* gene regulation was proven by the upregulation of numerous *var* genes and the loss of monoallelic *var* gene expression that occurred when either of two Sir2 histone deacetylase genes were disrupted [41], [48].

Alternative histones can replace canonical histones through ATP-dependent deposition to create structurally and functionally specialized chromatin domains [49]. While the importance of histone modifications to *P. falciparum* gene regulation is apparent, the role of alternative histones has not yet been investigated. H2A.Z is an H2A variant, which is essential for viability in most organisms apart from yeast and has been highly conserved through evolution [50], [51], [52], [53]. H2A.Z has been implicated in the regulation of very diverse processes such as heterochromatin formation, chromosome stability and segregation, proliferation and transcriptional activation or repression [49]. Its role in transcriptional regulation has been clearly established across species, but the underlying mechanisms are not well understood and reported effects of H2A.Z deposition are contradictory. Genome wide analyses consistently revealed that H2A.Z is enriched in nucleosomes near the transcriptional start site (TSS) in RNA polymerase II promoters [54], [55]. In human cells and in the protist parasite *Toxoplasma gondii*, H2A.Z enrichment at promoters (and enhancers in humans) correlates with gene activity [54], [56], whereas in yeast H2A.Z was found to occupy the promoters of active as well as poised genes from where it is lost with active transcription [55], [57].

In this study, we provide the first characterization of the alternative histone H2A.Z in *P. falciparum.* Using immunofluoresecence analysis (IFA), co-immunoprecipitation (Co-IP) and chromatin immunoprecipitation (ChIP) in conjunction with quantitative real time PCR (qPCR), we show that PfH2A.Z is enriched in the promoter of a set of developmentally regulated genes in the euchromatin compartment. In these genes promoter occupancy of PfH2A.Z does not correlate with transcription levels, suggesting that presence of PfH2A.Z is important for providing a transcriptionally competent chromatin structure at the promoter but does not directly relate to promoter activity. In *var* genes by contrast, PfH2A.Z promoter occupancy is strongly associated with transcriptional activity, as PfH2AZ is periodically enriched in the active *var* gene and is depleted in silent *var* genes, which is consistent with our IFA evidence that H2A.Z is absent from the subtelomeric heterochromatin compartment. This balance is distorted in cells in which the histone deacetylase Sir2A has been disrupted. Thus, these data suggest that *var* gene silencing requires expulsion of PfH2A.Z from the *var* promoter and that this involves histone deacetylation.

## Results

### The *P. falciparum* H2A.Z variant

The *P. falciparum* H2A.Z variant (PfH2A.Z) is encoded by the gene PFC0920w [58]. Sequence alignments show that PfH2A.Z shares 56.4% amino acid identity with the *S. cerevisiae* H2A.Z variant Htz1 and 67.3% identity with the human and mouse H2A.Z proteins (Figure S1), whereas conservation between other organisms was reported to be 70%–90% [59]. The major divergences affect the extended and highly charged N-terminus of PfH2A.Z, which is characterized by an accumulation of lysine residues and a three-fold repetition of the peptide sequence GGKV (position 9–20). Mass spectrometric evidence indicates that the N-terminus of PfH2A.Z can be acetylated in seven lysine residues, which would partially neutralize the positive charge [60]. In loop 1, two threonine residues implicated in mediating H2A.Z/H2A.Z self-interactions and prohibiting dimerization with H2A [61] are changed to isoleucine and serine (PfH2A.Z Ile 65 and Ser 66), respectively, and a conserved positively charged residue is substituted by aspartic acid (PfH2A.Z Asp 68) introducing a negative charge in this region. In the C-terminal docking domain all amino acids critical for interaction with the H3/H4 dimer are conserved [61].

### Developmental expression of PfH2A.Z during the asexual life cycle

**Figure 1:**
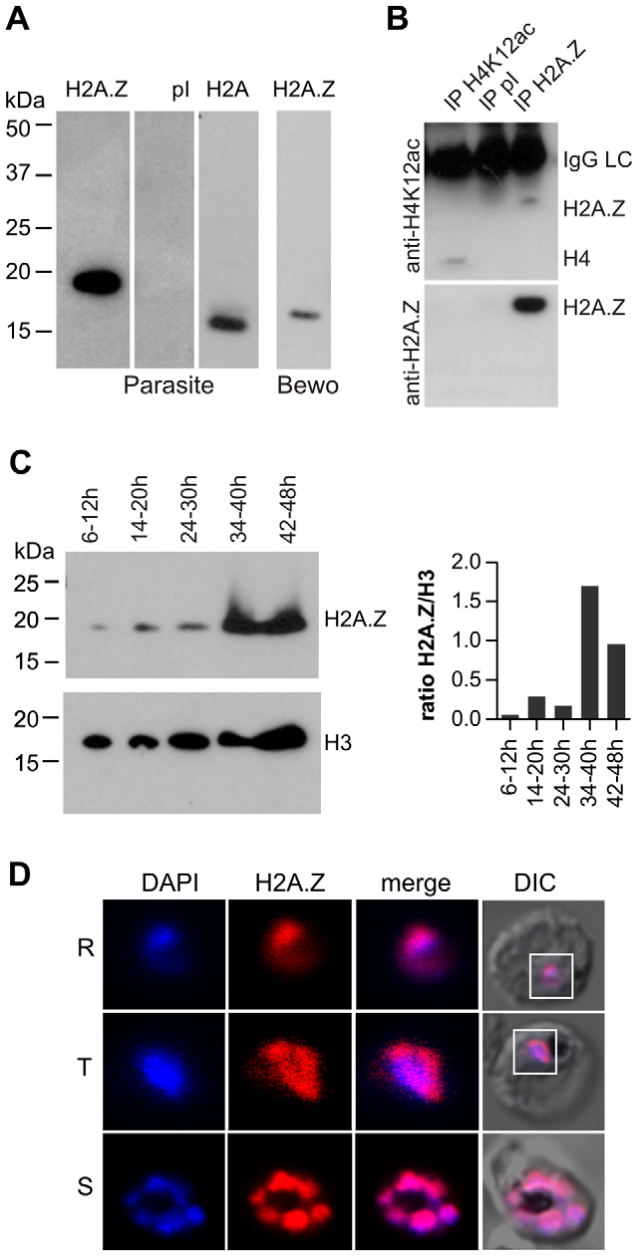
PfH2A.Z is expressed in the nucleus throughout asexual differentiation. Full length PfH2A.Z was expressed as a GST-fusion protein in *E. coli* and used to immunize rabbits. (A) Specificity of antisera. Parasite extracts were separated by SDS-PAGE and analysed by western blot. Anti-PfH2A.Z antiserum specifically reacted with PfH2A.Z at 18 kDa in parasite extracts and did not cross-react with H2A at 15 kDa (1^st^ panel). Pre-immune serum (pI) does not show any reactivity (2^nd^ panel). Anti-H2A antiserum specifically detects H2A migrating at 15 kDa (3^rd^ panel). Anti-PfH2A.Z detects human H2A.Z in BeWo cell lysate (4^th^ panel). (B) Anti-PfH2A.Z immunoprecipitates acetylated PfH2A.Z. Upper panel: Anti-H4K12ac antibody labels immunoprecipitated PfH2A.Z (lane 3). Anti-H4K12ac recognises an acetylated epitope present in both H4 and PfH2A.Z. Anti-H4K12ac IP (lane 1) was performed as a positive control and shows precipitation of a band corresponding to H4. No bands are apparent after IP with pI serum (lane 2). The IgG light chain (IgGLC) from the precipitating antibodies is also detected by the secondary antibody. Lower Panel: western blot reprobed with anti-PfH2A.Z confirms specificity of the immunoprecipitation. (C) Western blot analysis across the asexual life cycle demonstrates expression of PfH2A.Z and H3 in all stages. In comparison to H3, PfH2A.Z protein expression peaks in parasites 34–40 hours post-invasion which corresponds to late trophozoites/early schizonts. The ratio of H2A.Z/H3 signal in the western blot was determined by densitometry and is presented in a bar graph. (D) Nuclear localization of PfH2A.Z is shown by indirect immunofluorescence analysis and confocal microscopy of fixed 3D7 parasites using anti-PfH2A.Z antibodies. DNA was visualized with DAPI. R  =  ring stage, T  =  trophozoite stage, S  =  schizont stage. DIC  =  differential interference contrast.

To analyse PfH2A.Z, antiserum against recombinant full length PfH2A.Z was generated. The serum specifically recognizes PfH2A.Z but not H2A and cross-reacts with human H2A.Z, which has a different N terminal acetylation pattern, indicating reactivity of the antibody with the non-acetylated, conserved C-terminal domains (Figure 1A). We further showed that anti-PfH2A.Z immunoprecipitates both acetylated and non-acetylated forms of PfH2A.Z because antibodies specific for an acetylated peptide present in the PfH2A.Z and H4 N-termini [61] labels immunoprecipitated PfH2A.Z by Western Blot (Figure 1B).

We found that PfH2A.Z is present throughout the asexual life cycle (Figure 1C & D). In comparison to the canonical histone H3, PfH2A.Z abundance increases significantly in the schizont stage (Figure 1C). This is consistent with an increase in PfH2A.Z mRNA observed in schizonts [58]. We confirmed that PfH2A.Z is localized in the nucleus in all asexual stages by immunofluorescence analyses (IFA) and confocal microscopy. An area stained with the DNA dye DAPI but devoid of PfH2A.Z labelling was consistently observed, indicating that PfH2A.Z is enriched towards one side of the nucleus (Figure 1D). 3D reconstruction verified this polarized localization of PfH2A.Z (Video S1) and transgenic *P. falciparum* ectopically expressing PfH2A.Z-GFP fusion proteins corroborated the sub-nuclear distribution of PfH2A.Z (Figure S2). A similar cap-like pattern has previously been reported for the euchromatic histone mark H3K4me3 [62].

### PfH2A.Z is present in the euchromatin compartment

**Figure 2:**
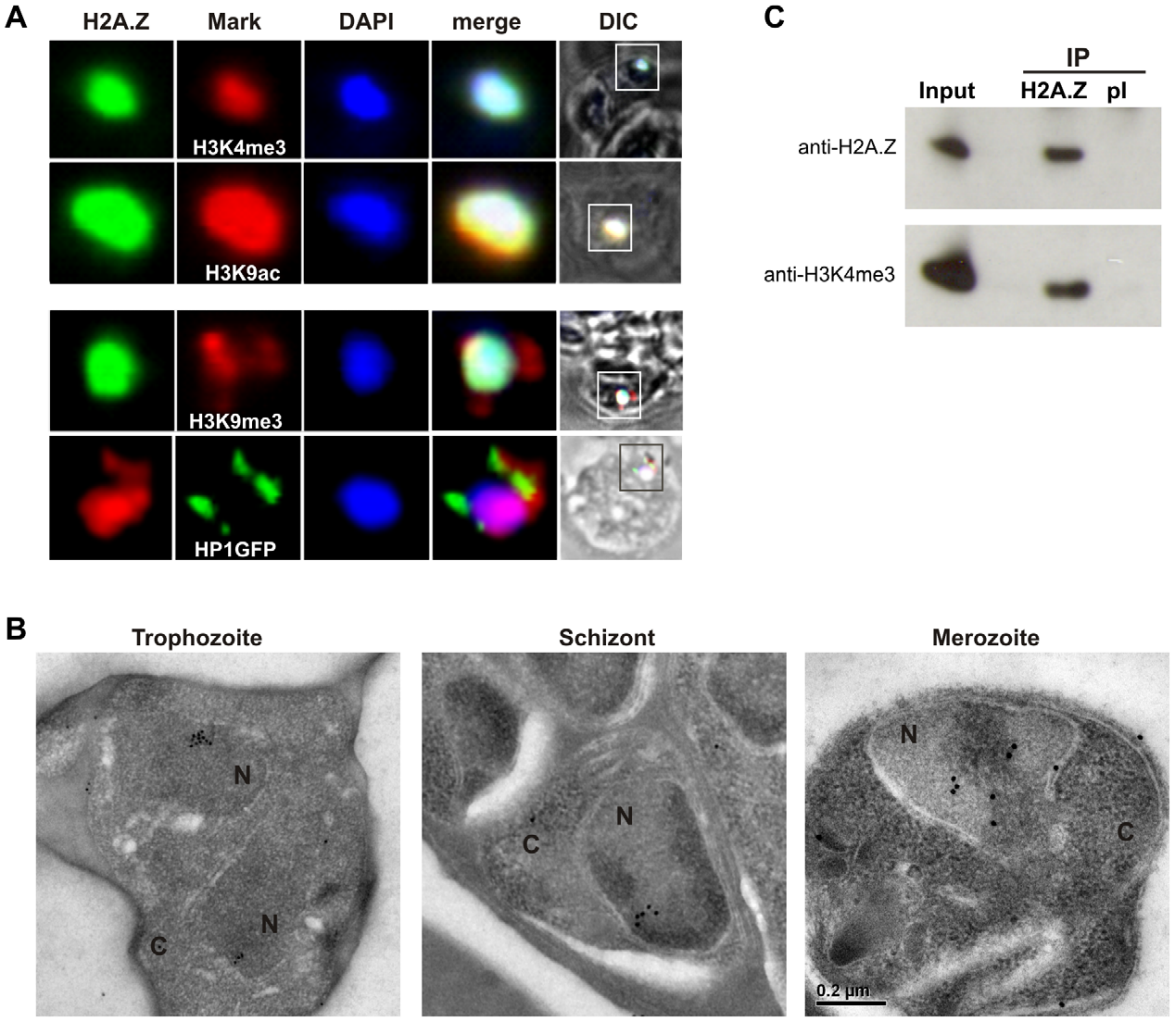
PfH2A.Z is present in the euchromatin compartment. (A) Colocalization analysis shows PfH2A.Z overlap with the euchromatin marks H3K9ac and H3K4me3 but not with the subtelomeric heterochromatin marks H3K9me3 and HP1. PfH2A.Z, H3K4me3, H3K9ac and H3K9me3 were detected with specific antibodies. (B) Immunoelectronmicroscopy with anti-PfH2A.Z antibodies indicates nuclear compartmentalization of PfH2A.Z. N  =  nucleus, C  =  cytoplasm. (C) Immunoprecipitation (IP) of mononucleosomes with anti-PfH2A.Z (H2A.Z) co-precipitates PfH2A.Z and H3K4me3. IP with pre-immune serum (pI) in parallel verified the specificity of the precipitation. Precipitated material was separated by SDS-PAGE and analysed by western blot. 3D7 whole parasite lysate was used as a positive control (Input). Western blots were probed with anti-PfH2A.Z and anti-H3K4me3 antibodies, respectively.

H2A.Z has been shown to contribute to diverse biological processes associated with different chromatin compartments, such as gene activation and poising [63], [64], [65], [66], chromosome segregation [67], [68] and heterochromatin structure [69], [70], [71], [72], [73]. To investigate the chromatin association of PfH2A.Z we performed co-localization experiments with well-characterized chromatin marks. Double staining showed good overlap between PfH2A.Z and the euchromatin marks H3K4me3 and H3K9ac (Figure 2A), which are enriched across the *P. falciparum* genome [22]. In contrast, PfH2A.Z staining was distinct from the subtelomeric heterochromatin marks H3K9me3 and HP1 (Figure 2A). Consistent with these results, immunoelectron microscopy indicated that PfH2A.Z was not restricted to the nuclear periphery, where inactive subtelomeric and internal *var* genes cluster. However, its distribution appeared concentrated in certain subnuclear compartments, frequently at the border of the electron lucent and electron dense nuclear material that is presumed to represent euchromatin and heterochromatin, respectively (Figure 2B) [38].

In mammalian cells it was shown that H2A.Z-containing nucleosomes preferentially carry the euchromatic mark H3K4me3 [73], [74]. Consistent with this, we found by co-immunoprecipitation experiments with anti-PfH2A.Z that mononucleosomes containing PfH2A.Z are highly enriched in H3K4me3 (Figure 2C). Together, these data support that PfH2A.Z is functionally linked to euchromatin but is largely depleted from subtelomeric heterochromatin.

### PfH2A.Z is enriched near the transcription start site (TSS) of genes irrespective of transcriptional status

To identify the genomic target sites of PfH2A.Z and investigate how the presence of PfH2A.Z correlates with transcriptional activity, we performed chromatin immunoprecipitation (ChIP) followed by quantitative PCR in ring stage parasites, trophozoites and schizonts. H2A.Z is enriched in nucleosomes surrounding the transcription start site (TSS) in other organisms, therefore at least two quantitative (q) PCR reactions were performed for each gene to amplify regions near the predicted TSS upstream of the start codon (ups) as well as in the open reading frame (orf).

**Figure 3:**
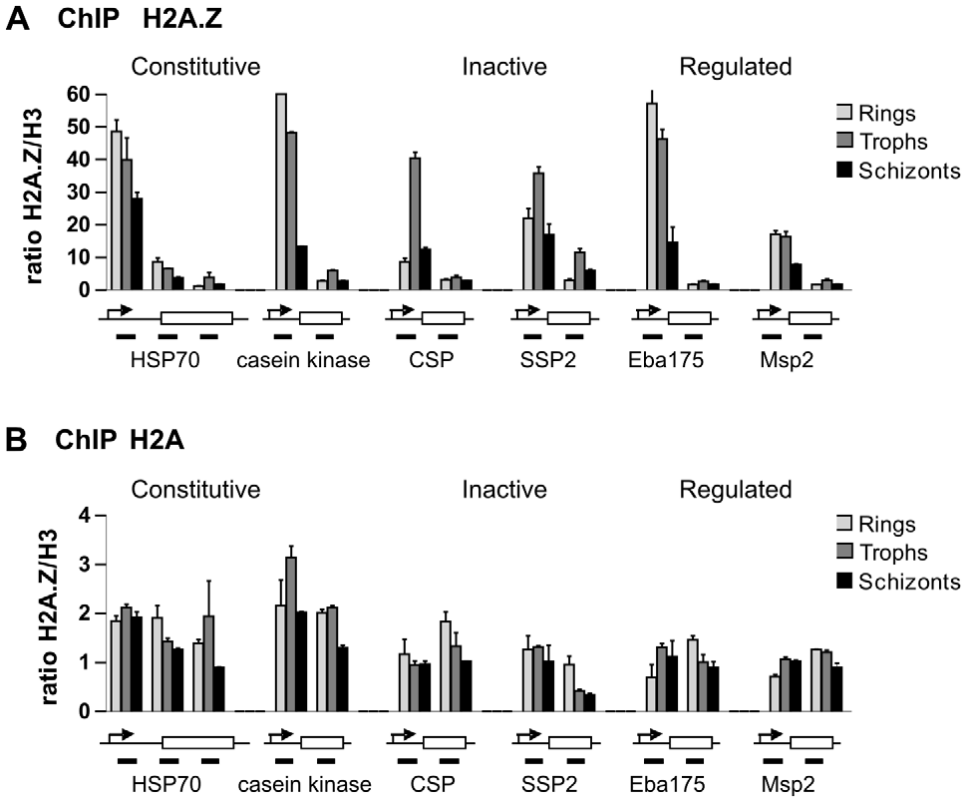
ChIP analysis of genomic PfH2A.Z distribution shows enrichment near the transcription start site (TSS). ChIP was performed in ring, trophozoite and schizont stage parasites with antibodies against PfH2A.Z, H2A, H3 and non-immune control antibodies. Real time qPCR was performed targeting sequences near the TSS and in the open reading frame of various genes characterized by different expression profiles. Enrichment was calculated and the data are presented as ratio over H3 to correct for differences in nucleosome density in the inter- and intra-genic regions. Error bars represent standard deviation from two technical replicates. One representative experiment out of three biological replicates is shown. Amplified regions are depicted in the gene models under each graph. Arrows depict the TSS, and boxes the protein coding sequence. (A) Enrichment of PfH2A.Z/H3 is observed near the TSS in all investigated genes in all three stages. (B) Enrichment of H2A/H3 is equivalent near the TSS and in the coding regions of genes in all three stages.

Our results show that PfH2A.Z is enriched in all three stages in the promoter regions of candidate genes that are differentially regulated throughout the life cycle (Figure 3A, S3A). In contrast to PfH2A.Z, H2A showed equal distribution in the promoter and the open reading frame of the investigated genes (Figure 3B). In rings, promoter enrichment of PfH2A.Z was highest in genes that are constitutively expressed (e.g. HSP70 and casein kinase) or induced during asexual intra-erythrocytic differentiation (e.g. schizont genes MSP2 and Eba175), but also clearly apparent in silent genes (e.g. sporozoite genes CSP and SSP2). In trophozoites and schizonts, PfH2A.Z enrichment in the promoter showed similar levels across all genes.

To determine whether the level of PfH2A.Z promoter occupancy in the examined genes correlated with gene expression, mRNA levels were quantified by q-RT-PCR (Figure S4). The ups/orf ratio of PfH2A.Z enrichment was determined and plotted against the relative expression levels. No significant correlation between PfH2A.Z promoter occupancy and transcription level could be observed at any stage (Spearman correlation, p>0.5).

To further investigate the relationship between PfH2A.Z and euchromatic and heterochromatic histone marks, ChIP was performed in parallel for PfH2A.Z as well as H3K4me3, H3K9ac and H3K9me3. PfH2A.Z and the two euchromatic marks, H3K4me3, H3K9ac all showed significant enrichment in the ups region when compared to the orf in ring and schizont stage parasites, in contrast the levels of the heterochromatic mark H3K9me3 were the same in ups and orf (Figure S5). This is consistent with previous work on the histone marks [22] and supports our finding that PfH2A.Z and H3K4me3 are present in the same nucleosomes (Figure 2C). Furthermore, we found that the PfH2A.Z enrichment level in the upstream region of genes positively correlates with both euchromatic marks (P<0.0001), whereas a negative correlation was evident between PfH2A.Z and H3K9me3 (p<0.0001) (Figure S6).

Together, these results demonstrate that PfH2A.Z is enriched near the TSS in genes, independently of their transcriptional activity, and that PfH2A.Z enrichment near the TSS correlates with enrichment of the euchromatin marks H3K4me3 and H3K9ac.

### PfH2A.Z enrichment in the *var* gene promoter is developmentally regulated

The *P. falciparum* histone deacetylases Sir2A and Sir2B silence subtelomeric and central *var* genes [41], [48], [75] and in *S. cerevisiae* H2A.Z antagonises subtelomeric gene silencing by Sir2 [53]. Therefore we investigated whether PfH2A.Z was involved in the transcriptional control of *var* genes. We harvested chromatin and RNA from a parasite culture that had been selected for the expression of a single *var* gene encoding VAR2CSA by panning on chondroitin sulphate A (CSA). To map the position of PfH2A.Z along the *var2csa* gene, we designed seven qPCR reactions spanning this *var* locus. The TSS of *var2csa* has previously been mapped to −1475 bp upstream of the start codon in the FCR3 parasite line [34] and is predicted to be located at approximately −1200 bp in 3D7 (PlasmoDB). Three primer pairs amplified regions in the non-coding upstream region (-1500 bp, −1000 bp, −575 bp), and four primer pairs targeted areas along the coding region.

**Figure 4:**
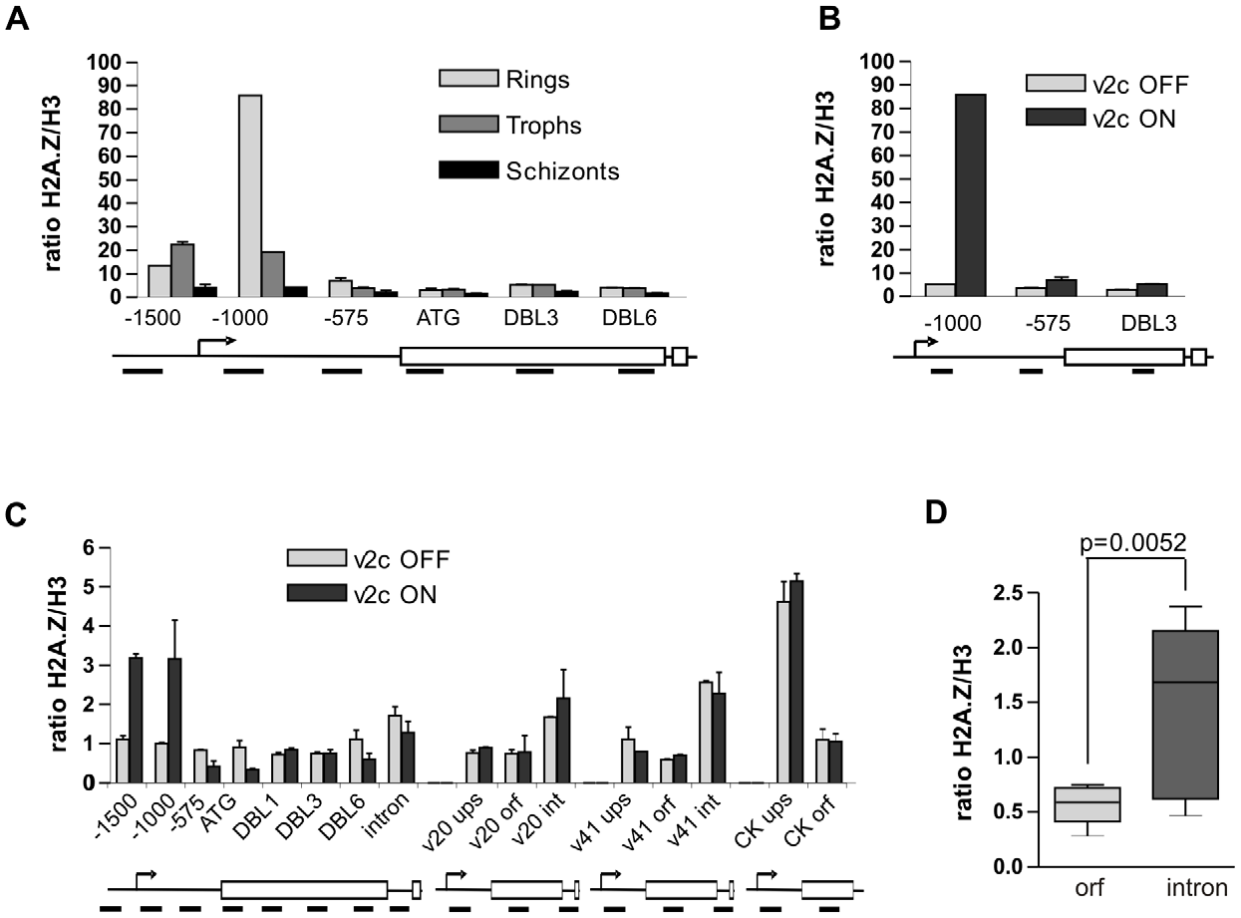
PfH2A.Z is enriched in the active *var* upstream region and in *var* introns. (A) ChIP analysis of PfH2A.Z distribution along the *var2csa* gene throughout differentiation in 3D7 parasites selected for *var2csa* expression by CSA panning. PfH2A.Z is enriched at −1000 and −1500 bp upstream of the ATG in ring and trophozoite stages, but not in schizonts. These positions correspond to nucleosomes surrounding the TSS. One representative experiment out of four biological replicates is shown. (B) PfH2A.Z occupancy in ring stage parasites expressing *var2csa* (var2CSA ON) or not (var2CSA OFF). PfH2A.Z enrichment near the TSS is only observed when *var2csa* is expressed. (C) PfH2A.Z distribution along *var2csa,* PFL0020c (var20), PF08_0141 (var41) and caseine kinase (CK) as a control. The transcribed *var2csa* locus (var2CSA ON) shows enrichment of PfH2A.Z around the TSS and moderately in the intron. In silent *var* genes, PfH2A.Z is exclusively enriched in the intron. Amplified regions are depicted in the gene models under each graph. ups  =  upstream, orf  =  open reading frame, int  =  intron. Error bars represent standard deviation from two technical replicates. (D) PfH2A.Z is significantly enriched in the *var* intron as compared to the orf (Mann-Whittney, N = 10, p = 0.0052). Intron data are compiled from three different experiments of ten different *var* genes.

ChIP analysis showed that in ring stage parasites, when *var2csa* transcription peaks (Figure S4) [76], PfH2A.Z is strongly enriched in the areas flanking the predicted TSS (−1500 and −1000 bp), but not further downstream (−575 bp) or within the open reading frame (ATG, DBL3, DBL6) (Figure 4A, S3B). As the parasites progress through the trophozoite to the schizont stage *var* transcription declines and PfH2A.Z enrichment around the TSS decreases. No enrichment of PfH2A.Z was detectable around the TSS of the *var2csa* gene at schizont stage. This stage-specific deposition of PfH2A.Z in the *var* gene promoter contrasts with the continuous presence of PfH2A.Z in the promoters of the limited number of other genes we analysed (Figure 3A) indicating that PfH2A.Z deposition differs in its temporal regulation in *var* genes.

To directly compare PfH2A.Z occupancy of an active and inactive *var* promoter we performed ChIP experiments on unselected 3D7 parasites that do not transcribe the *var2csa* gene (*var2csa* OFF), and *var2csa* expressing parasites (*var2csa* ON) at ring stage. Transcription levels were verified by q-RT-PCR (Figure S4). In contrast to the strong enrichment of PfH2A.Z in the active *var* promoter, the alternative histone was clearly not enriched when *var2csa* was not transcribed. These results demonstrate that PfH2A.Z promoter occupancy in *var2csa* strongly correlates with transcription (Figure 4B).

To verify our observation that PfH2A.Z occupancy is restricted to the active *var* TSS in a second *var* gene, 3D7 parasites were selected on ICAM1 and the gene PFL0020w was identified as the dominant transcript by q-RT-PCR and Northern Blot analysis (Figure S7 A, B). ChIP analysis across the gene confirmed increased PfH2A.Z occupancy near the PFL0020w TSS in ICAM1 selected parasites as compared to non-selected parasites at ring stage. In line with our results with *var2csa*, no enrichment was evident in schizonts (Figure S7 C, D).

### PfH2A.Z is enriched in the *var* intron

*Var* genes possess a second promoter, which is situated in the conserved intron. From this promoter, truncated sense and antisense transcripts are synthesized which are thought to contribute to heterochromatin structure and *var* gene silencing [77]. To investigate a possible association of PfH2A.Z with the *var* intron promoter, we designed primer pairs targeting the *var* introns and repeated the ChIP experiment in *var2csa* expressing and non-expressing ring stage parasites (Figure 4C). Consistent with the previous observations, PfH2A.Z was enriched around the TSS of the active *var2csa* gene (*var2csa* ON), but not the TSS of inactive *var2csa* (*var2csa* OFF) nor the TSS of the other two silent *var* genes PF08_0141 (*var41*) and PFL0020w (*var20*). In contrast to the upstream region, all three *var* genes were moderately enriched in PfH2A.Z in the introns (Figure 4C). This was also evident in the ICAM selected parasite line (Figure S7 C&D). Analysis of intron/orf pairs from ten different *var* genes confirmed that the enrichment of PfH2A.Z in the intron was statistically significant (Figure 4D).

### PfH2A.Z deposition is maintained at the promoters of active *var* genes in mature Sir2AKO, but not Sir2BKO parasites

**Figure 5:**
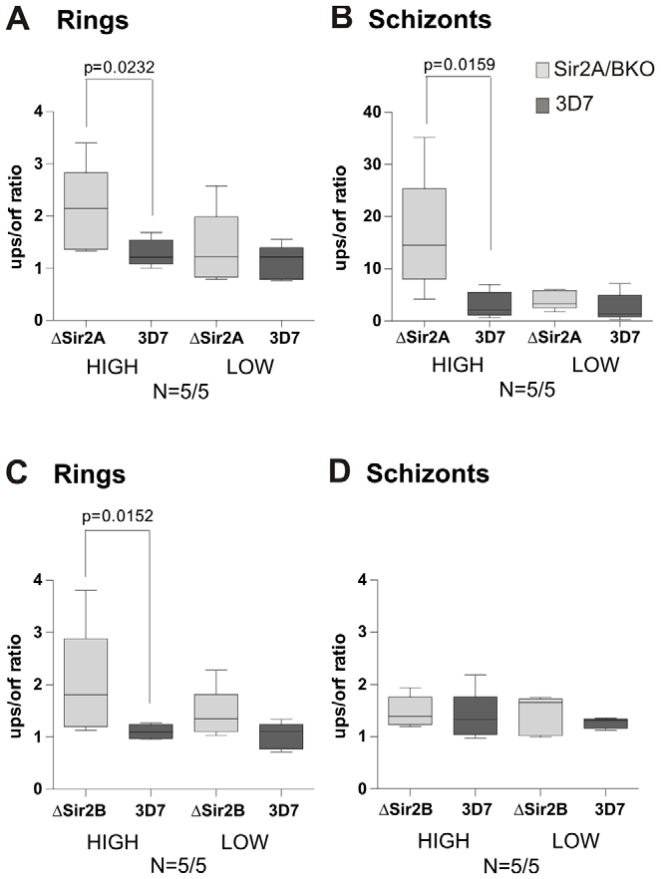
PfH2A.Z occupancy in the active *var* gene upstream region is maintained at schizont stage in ΔSir2A but not ΔSir2B parasites. ChIP was performed in ring (A, C) and schizont stage (B, D) parasites of 3D7, 3D7ΔSir2A and 3D7ΔSir2B lines. One out of two biological replicates is shown. Real-time qPCR was performed in upstream and coding regions of different sets of five *var* genes that were expressed at high or low levels in 3D7ΔSir2A or in 3D7ΔSir2B, respectively. The ratio between PfH2A.Z enrichment in the upstream and coding sequence was determined (ups/orf ratio). Knock out parasites are presented in light grey, wild type 3D7 parasites in dark grey. Shown is the median boxed with 25^th^ and 75^th^ percentile and minimum/maximum values as whiskers. Non-parametric Mann-Whitney test was performed and significant differences are indicated. Analysed *var* genes were PF13_0003, MAL7P1.50, PF07_0050, PFD1005c and PFL1960w (ΔSir2A high); PF08_0141, PFD0020c, PFL0020w, PF08_0106 and PFD0995c (ΔSir2A low); PFL2665c, PFA0005w, PF13_0364, PFD0615c and PF07_0049 (ΔSir2B high); PFE0005w, PF07_0139, PFD1245c, PFD0020c and PFD0005w (ΔSir2B low).

Because H2A.Z may function as a barrier to prevent the spread of Sir2-mediated silencing in yeast [53] we further investigated the relationship between Sir2 and PfH2A.Z in the control of *var* gene expression in *P. falciparum*. ChIP and expression analyses were performed on ring and schizont stage parasites in which Sir2A or Sir2B had been disrupted (3D7Δsir2A and 3D7Δsir2B) [41], [48]. By q-RT-PCR we first monitored the expression profiles of all *var* genes in ring stages of both 3D7Δsir2 parasite lines (Figure S8). With the aim to understand how each Sir2 paralogue influences PfH2A.Z deposition and how this correlates with *var* transcription, we selected five *var* genes that were highly expressed and five *var* genes that were lowly expressed in 3D7Δsir2A parasites for further analysis, all of which had previously been shown to be regulated by Sir2A [41], [48]. We used the same strategy to choose ten *var* genes previously shown to be regulated by Sir2B [48] for analysis in 3D7Δsir2B. We then analysed PfH2A.Z deposition by ChIP and qPCR in upstream and coding regions of these *var* genes in knock out and wild type parasites. The ups/orf ratios were determined and compared between 3D7Δsir2A or 3D7Δsir2B and 3D7 parasites, respectively (Figure 5).

Although ups/orf ratios were generally quite low in ring stages, PfH2A.Z enrichment at *var* promoters was significantly greater in the highly expressed *var* genes in the 3D7Δsir2A and B lines than in the 3D7 control (Mann-Whitney, p =  0.0232 (Δsir2A) and p = 0.0152 (Δsir2B)) (Figure 5A & C). In schizonts, no enrichment of PfH2A.Z upstream of active Sir2B regulated genes was detected (Figure 5D). This result is consistent with a role of PfH2A.Z in active transcription of *var* genes. However, interestingly PfH2A.Z occupancy in the upstream region of *var* genes was maintained and even elevated at schizont stage in 3D7Δsir2A parasites at significantly higher levels than in 3D7 (Mann-Whitney, p = 0.0159) (Figure 5B), although *var* gene expression is down-regulated in mature 3D7Δsir2 parasites (data not shown) [48]. This result indicates a link between Sir2A and loss of PfH2A.Z and implicates PfH2A.Z with maintenance of the integrity of the heterochromatin/euchromatin boundary at Sir2A regulated loci.

To further investigate the atypical PfH2A.Z enrichment in Sir2A regulated *var* genes in 3D7Δsir2A schizonts we analysed by ChIP the relationship between enrichment of PfH2A.Z and H3K9me3 or H3K4me3, respectively (Figure S9). Similar to actively transcribed *var* genes in immature wildtype parasites (Figure S6), we found a positive correlation between PfH2A.Z and H3K4me3 enrichment (Spearman correlation, p = 0.0005) and a.negative correlation between PfH2A.Z and H3K9me3 (Spearman correlation, p = 0.0182), which was consistent with the previously described depletion of H3K9me3 in *var* genes upregulated in 3D7Δsir2A parasites [35].

## Discussion

### The *P. falciparum* H2A.Z ortholog

H2A.Z is essential in many eukaryotes including *Trypanosoma brucei*, *Tetrahymena thermophilus*, *Drosophila*, *Xenopus*, and vertebrates [50], [52], [59], [78], [79] and is one of the structurally most conserved histones throughout evolution [80]. However, *Plasmodium* H2A.Z differs significantly from its orthologues, particularly in its extended N-terminus which contains seven lysine residues that can be acetylated [58], [60], as opposed to five in humans and four in yeast. In *T. thermophilus* the N-terminus of H2A.Z has been implicated in directly interacting with the DNA [81]. Acetylation of at least one lysine residue is essential for viability, and probably acts by reducing the positive charge and thereby weakening H2A.Z–DNA interactions [82]. Interestingly, *T. thermophilus* H2A.Z encodes a repeated GGK motif similar to the one observed in PfH2A.Z, suggesting that a similar mechanism may apply for regulating PfH2A.Z-DNA interactions in the malaria parasite.

By western blot we observed increased PfH2A.Z abundance in the schizont stage relative to H3. Interestingly, PfH2A.Z accumulation coincides with expression of the ATP-dependent chromatin remodeling factor PfSwr1 (unpublished data), orthologues of which mediate the post-replicative H2A.Z incorporation into nucleosomes in yeast and humans [83], [84], [85]. The *Plasmodium* genome becomes densely packed with nucleosomes in the late schizont stages [24], and the concurrent increase in PfH2A.Z may reflect a rising requirement for the alternative histone in the intergenic regions. H2A.Z facilitates intra-molecular folding of nucleosomal arrays into a 30 nm fibre [86], so it may play a role in chromatin condensation in the mature schizonts.

### PfH2A.Z and genes in the euchromatin compartment

Enrichment of H2A.Z in RNA polymerase II promoters has been conserved through the evolution of eukaryotes as diverse as yeast, humans and the protist parasites *T. brucei* and *T. gondii* [29], [53], [54], [55], [56], [57], [78], [87], [88]. Our ChIP analysis showed that *P. falciparum* conforms to this pattern, with an enrichment of PfH2A.Z, but not H2A, in the upstream regions of genes (Figures 3, 4). Consistent with this finding, we co-precipitated PfH2A.Z and H3K4me3 (Figure 2), which is enriched in 5′-upstream regions of several organisms including *P. falciparum* [22], [54], [89]. Our study also indicates that the pattern of PfH2A.Z occupancy at euchromatic gene promoters remains relatively stable throughout the IDC (Figure 3), in contrast to the reported fluctuations in levels of the transcriptionally permissive histone modifications H3K4me3 and H3K9ac [22]. Further substantiating an association of PfH2A.Z with promoter architecture, we showed that PfH2A.Z occupation in upstream regions correlates with H3K4me3 and H3K9ac enrichment (Figures S5 & 6).

With the exception of *var* genes, PfH2A.Z enrichment did not correlate with mRNA levels in our experiments. A global enrichment of H2A.Z at active and inactive promoters has also been observed in yeast [55], whereas some other studies reported a negative correlation with transcription and proposed that H2A.Z poises inducible silent genes for activation and is subsequently evicted during transcription [29], [57], [88]. In humans, H2A.Z is either present in active gene promoters in differentiated cells [54], [90] or marks poised gene promoters in hematopoietic stem cells [91]. These conflicting results demonstrate that the role of H2A.Z in transcription is very complex and the underlying mechanisms remain enigmatic.

In both yeast and humans H2A.Z assists in RNA polymerase II recruitment [90], [92]. Components of the preinitiation complex are pre-assembled in some erythrocytic stage *P. falciparum* promoters regardless of gene activity [93], similar to the PfH2A.Z enrichment at the TSS shown here. Thus PfH2A.Z may contribute to the open chromatin structure necessary for preinitiation complex formation. But how could PfH2A.Z modulate gene activity despite global occupancy at promoters? Mass spectrometric evidence that PfH2A.Z can be heavily acetylated at multiple lysine residues in the N-terminus suggests that the neutralization of positive charges by lysine acetylation may facilitate an open chromatin structure making the DNA more accessible [58], [60]. In our experiments, total PfH2A.Z occupancy was monitored because our antiserum did not differentiate between acetylated and non-acetylated forms of PfH2A.Z (Figure 1). Acetylation may thus provide a functional switch necessary to promote transcription, as has been suggested for yeast and humans [64], [66], [94], [95]. Histone acetylation normally promotes gene activation, whereas sumoylation and ubiquitination are post-translational modification associated with recruitment of histone deacetylase complexes and transcriptional repression [96], [97]. PfH2A.Z has six potential sumoylation sites [98] and may also be ubiquitinated in the C-terminal domain, although this has not yet been shown. In mammalian cells, mono-ubiquitinated H2A.Z is enriched in the facultative heterochromatin that constitutes the inactive X-chromosome [73]. Thus, acetylation, ubiquitination and sumoylation represent interesting candidate regulators of PfH2A.Z function at promoters.

In *Arabidopsis thaliana* and yeast it has recently been shown that H2A.Z mediates a thermo-sensory response and facilitates differentiation processes by regulating transcription [99]. This is thought to be due to reduced DNA wrapping of H2A.Z containing nucleosomes at higher temperatures, resulting in a relaxed chromatin structure that permits of transcription. It is tempting to speculate that PfH2A.Z may function as a similar physical switch to control gene expression in response to temperature change, for example during fever or as *P. falciparum* is transmitted between its two hosts.

Apart from PfH2A.Z, *P. falciparum* encodes two other histone variants, H2Bv and H3.3 [58], which have previously been implicated in transcriptional regulation. H2Bv is a variant unique to protist parasites and has so far only been characterized in *T. brucei* and *T. gondii* [56], [78], [87]. In both parasites H2Bv pairs with H2A.Z. While H2A.Z/H2Bv nucleosomes are enriched in the promoters of active genes in *T. gondii* [56], they showed a global association with PolII transcription start sites in *T. brucei* [87]. Nucleosomes containing H2A.Z and H3.3 have been reported to be less stable than nucleosomes containing H2A.Z and canonical H3 in humans [100], and nucleosomes composed of both variant histones are present in active promoters, enhancers and insulators. It was postulated that instability of H2A.Z/H3.3 composite nucleosomes at the TSS might facilitate access to transcription factors [101]. H3.3 differs only by three amino acids from canonical H3 so the antibodies directed against H3 in this study precipitated both variants. The functional cooperation of alternative histones in *P. falciparum* gene regulation will be an interesting field of future research.

### PfH2A.Z and *var* genes

In many organisms, including *Plasmodium,* subtelomeric genes are subject to special mechanisms of gene regulation. This occurs via the unique subtelomeric heterochromatin which provides a specialized architecture to control variegated expression of gene family members (reviewed in [102], [103]). A link between H2A.Z and subtelomeric gene regulation has been established in *S. cerevisiae* as disruption of the H2A.Z orthologue htz1 results in the down regulation of many subtelomeric genes [53]. Consistent with its proposed role as an anti-silencing factor for subtelomeric genes in yeast, we found PfH2A.Z to be enriched in the subtelomeric *var2csa* and *pfl0020w* promoters when these genes were active, but not when they were silent (Figures 4, S7). This was supported by significant enrichment of PfH2A.Z in active *var* genes in the two Sir2 KO lines (Figure 5). Thus, PfH2A.Z is a novel component contributing to the promoter architecture of active *var* genes together with the euchromatin factors H3K4me3 and H3K9ac [34]. In contrast to the other genes we examined, *var* genes exhibited significant temporal modulation in PfH2A.Z enrichment at the promoter. Loss of PfH2A.Z from the *var* promoter is observed from the trophozoite stage on, which is when DNA replication begins, and thus is consistent with S-phase dependent silencing of *var* genes [43]. While canonical histones are deposited into chromatin at the replication fork, deposition of histone variants can occur post-replication by ATP-dependent enzyme complexes. This allows dynamic changes to the chromatin structure at the promoter during differentiation (reviewed in [49]). Based on this knowledge we speculate that PfH2A.Z is lost from the *var* promoter during DNA replication, possibly due to limited stability of PfH2A.Z containing nucleosomes, and is deposited at the *var* promoter in rings. The loss of PfH2A.Z from the active *var* gene promoter upon temporal silencing suggests that, unlike in subtelomeric genes in yeast [57], [104], PfH2AZ is not involved in epigenetic memory of *var* genes.

Our ChIP and qPCR experiments along the entire *var* gene revealed that PfH2A.Z enrichment peaks at positions directly surrounding the predicted TSS of the active *var* promoter. This pattern is consistent with yeast where H2A.Z is highly enriched in the two nucleosomes surrounding a nucleosome free-region at the TSS, and with humans where it is restricted to the two nucleosomes in the −1 and +1 position as well as at the TSS itself [55], [101]. The distinctive pattern of PfH2A.Z enrichment we observed within the *var* intron corroborates the existence of a promoter-like chromatin structure at this site. The *var* gene intron contains a bi-directional promoter from which sense and antisense transcripts are synthesized in both active and inactive *var* genes during late erythrocytic stages, indicating that this mechanism might function in the control of transcriptional timing rather than monoallelic expression [38], [77]. The impact of the regulatory activity of the *var* gene intron upon local chromatin structure has been previously indicated through nucleosome depletion within the intron [24], [28]. While PfH2A.Z occupancy at the *var* upstream promoter could only be detected when *var2csa* was actively transcribed, enrichment at the conserved intron promoter occured in both active and inactive *var* genes. This is consistent with the previously observed lack of correlation between activity of the *var* intron promoter and of the upstream *var* promoter [38], [77].

### PfH2A.Z and Sir2

In yeast, H2A.Z acts as a boundary element that antagonizes the spread of Sir2-mediated heterochromatin into euchromatic areas [53], [105]. This is consistent with our findings by IFA, CoIP and ChIP revealing that PfH2A.Z is associated with regions of euchromatin but is absent from the heterochromatin compartment that is characterized by H3K9me3 and HP1 deposition and contains silent *var* genes (Figures 2, 3, 4). Interestingly, our electron microscopy analysis suggests enrichment of PfH2A.Z at the border between electron light and electron dense nuclear material (Figure 2), which has previously been interpreted to represent eu- and heterochromatin, respectively [38]. This raises the prospect that PfH2A.Z may also form a barrier to the spread of Sir2-mediated heterochromatin. This is supported by our finding that in 3D7Δsir2A parasites PfH2A.Z is enriched in the upstream region of *var* genes that are highly transcribed not only when the *var* genes are active in ring stages but also when they are silent in mature schizonts (Figure 5 & [41], [48]). In contrast PfH2A.Z is enriched in the upstream region of highly expressed *var* genes in 3D7Δsir2B parasites only while the *var* genes are active in the ring stages. Sir2A-regulated *var* genes occupy the heterochromatin/euchromatin boundary at both chromosome internal and subtelomeric clusters; in contrast Sir2B regulates the most telomere proximal upsB type *var* genes that are separated from the rest of the chromosome by the Sir2A regulated subtelomeric upsA *var* genes [41], [48]. This raises the intriguing possibility that antagonism between Sir2A and PfH2A.Z is involved in maintaining the heterochromatin/euchromatin boundary but Sir2B is not. Future experiments testing the enrichment of PfH2A.Z at boundary sites using ChIP will address this question.

**Figure 6:**
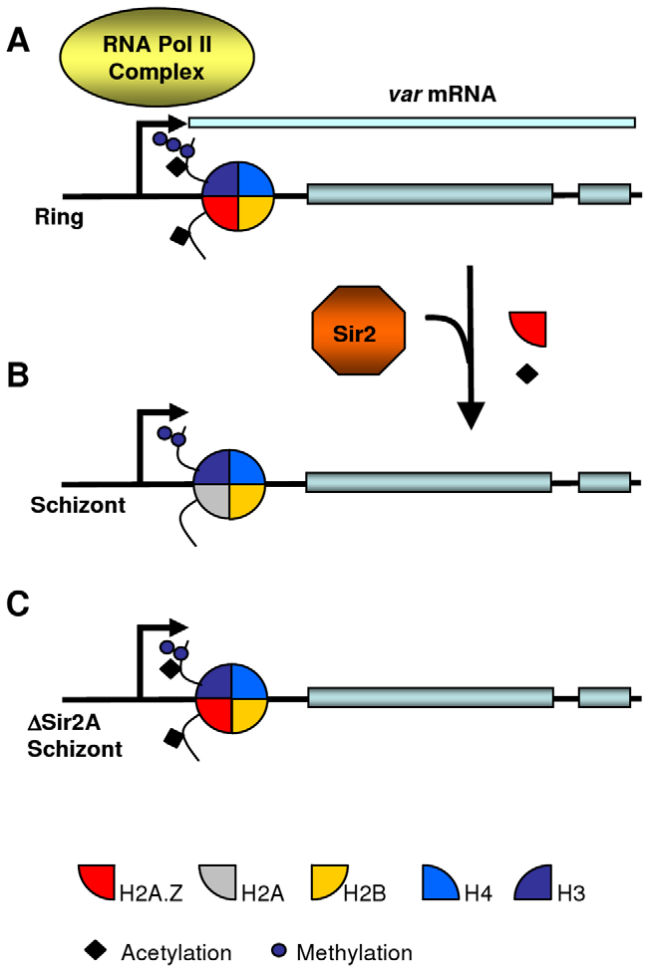
Hypothetical model for PfH2A.Z dynamics at the *var* transcription start site. (A) In rings, H3K4me3, H3K9ac and PfH2A.Z are present at the TSS of the *var* gene that is actively transcribed by the RNA PolII complex. (B) In schizonts, the active *var* gene is in a silent state poised for transcription in the next cycle. H3K4me3 at the promoter is demethylated to H3K4me2 [34]. H3K9 is deacetylated and PfH2A.Z removed, which might be mediated by the histone deacetylase Sir2A. (C) In parasites in which Sir2A is disrupted, H3K9ac and PfH2A.Z are maintained at the poised *var* promoters in schizont stages.

Possibly PfSir2A is not only involved in maintaining *var* gene silencing in heterochromatin by removing activating histone acetylations such as H3K9ac [35], but also assists in the temporary expulsion of PfH2A.Z from the active *var* promoter in mature parasites (Model in Figure 6). This could occur indirectly through recruitment of the ATP-dependent chromatin remodeling machinery responsible for histone variant exchange, or directly through Sir2-mediated deacetylation of PfH2A.Z. Sir2 plays such a direct role in regulating H2A.Z levels in human myocytes where over-expressed Sir2 deacetylates H2A.Z which in turn leads to ubiquitination and proteasome-dependent degradation of H2A.Z [106].

### Conclusion

Epigenetic gene regulation contributes significantly to the transcriptional control of fundamental mechanisms such as differentiation and antigenic variation of the malaria parasite (reviewed in [107]). Here, we identify the alternative histone PfH2A.Z as a component of nucleosomes in the promoters of euchromatic genes, suggesting it may be involved in gene regulation. Our report provides the first description of histone variant exchange in *P. falciparum,* as we demonstrate a temporal modulation of PfH2A.Z occupancy in *var* genes. This mechanism is disturbed in parasites in which the histone deacetylase Sir2A is disrupted, suggesting a functional link between Sir2A and regulation of PfH2A.Z dynamics at the *var* promoter.

## Materials and Methods

### Parasite lines and culture

*P. falciparum* lines 3D7, 3D7ΔSir2A [41], 3D7ΔSir2B [41] and 3D7HP1GFP [33] were cultured in RPMI medium supplemented with 5% heat-inactivated human serum 0.25% albumax [108]. Synchronicity was maintained by weekly treatment with 5% sorbitol. Selection of var2CSA expressing parasites was performed by panning on plastic dishes coated with 50 µg/ml bovine trachea CSA (*Sigma*), as described previously [108].

### Recombinant protein expression and immunization

The full length PfH2A.Z (PFC0920w) sequence was amplified from 3D7 cDNA using oligonucleotides PfH2A.ZBamHIFor: 5′-GGGATCCGGATGGAAGTTCCAGGAAAAGT and PfH2A.ZEcoRIRev: 5′-GAATTCTTATTGAGCTGTTGGGGGAAGTG. The PCR product was cloned into pGEX-5X-3 (*GE Healthcare*). PfH2A.ZGST fusion protein was expressed in BL21 cells in Luria Broth and induced with 1mM IPTG. Bacteria pellets were resuspended in Bugsbuster reagent and benzonase (*Novagen*) and the soluble proteins were purified on GST-Bind Resin (*Novagen*). Antibodies were generated in rabbits by the Walter and Eliza Hall Institute Monoclonal Antibody Facility (*Bundoora, Vic. Australia*).

### Antibodies

Primary antibodies employed in ChIP assays in this study were rabbit anti-PfH2A.Z, rabbit anti-H3 (*Abcam* Ab1791), rabbit anti-H2A (*Millipore* 07-146), rabbit anti-H2B (*Abcam* Ab1790), non-immune rabbit IgG (*Abcam* Ab 46540) and pre-immune rabbit serum. Primary antibodies used for IFA co-localization and WB were rabbit anti-H3K9me3 (*Abcam* Ab8898), rabbit anti-H3K4me3 (*Millipore* 04-745) and rabbit anti-H3K9ac (*Millipore* 06-942). Secondary antibodies for IFA were goat anti-rabbit AlexaFluor488 or chicken anti-rabbit AlexaFluor594 (*Molecular Probes*). Secondary antibodies for WB were goat anti-rabbit HRP (*Invitrogen*).

### Parasite lysates for Western Blot analysis (WB)

Parasites were harvested in 8 hour intervals at 5–10% parasitemia. Lysates were generated by saponin lysis of cultures and extraction of the resulting parasite pellets with 2 x SDS PAGE loading buffer. Equivalents of 5×10^7^ IE were separated by SDS-PAGE on 10% Bis-Tris gels (*Invitrogen*) and analysed by Western Blotting as described previously [109].

### Immunofluorescence analysis (IFA)

IFA was performed on paraformaldehyde/glutaraldehyde-fixed cultures as described previously [110]. For co-localization studies, histone modifications were first labelled using chicken anti-rabbit AlexaFluor594 (1∶1000) (*Molecular Probes*) as a secondary antibody. PfH2A.Z was subsequently detected with affinity purified rabbit anti-PfH2A.Z (10 ng/µl) directly labelled using the Zenon Rabbit IgG labelling kit (*Invitrogen*) according to the manufacturers instructions. IE were mounted onto slides using ProLong antifade (*Invitrogen*), left overnight to cure and analysed with an Olympus FV1000 Confocal Laser Scanning Microscope and the FluoView software.

### Immunoelectron microscopy

Parasites were fixed in 1% glutaraldehyde for 1 h at 4°C, dehydrated in increasing ethanol concentrations, then embedded in LR Gold resin (*Electron Microscopy Sciences, Fort Washington, PA*). Ultrathin (90–100 nm) sections were cut using a Leica Ultracut R microtome, labeled with rabbit anti-PfH2A.Z and goat-anti-rabbit IgG conjugated to 12 nm colloidal gold (*Jackson ImmunoResearch Laboratories*). Sections were poststained with uranyl acetate and lead citrate and observed using a Philips CM120 BioTwin Transmission Electron Microscope.

### Chromatin immunoprecipitation (ChIP)

Chromatin was isolated at three time points during the intra-erythrocytic developmental cycle (IDC) from early ring (6–14 hpi), trophozoite (24–32 hpi) and schizont (36–44 hpi) stage parasites. Parasite cultures were cross-linked with 1% paraformaldehyde for 10 min at 37°C and the reaction subsequently quenched with 125 mM glycine. After one wash with PBS parasites were released from IE by saponin lysis, and nuclei were isolated by incubation for 30 min on ice in lysis buffer (10 mM Hepes pH 7.9, 10 mM KCl, 0.1 mM EDTA, 0.1 mM EDTA, 1 mM DTT, 1x EDTA-free protease inhibitor cocktail (*Roche*)) followed by dounce homogenization (Pestle B). 0.25% NP40 was added to the parasite suspension prior to homogenization. Nuclei were pelleted by centrifugation at 21,000×g for 10 min at 4°C and resuspended in SDS lysis buffer (1% SDS, 10 mM EDTA, 50 mM Tris pH 8.1, 1 x EDTA-free protease inhibitor cocktail). Chromatin was sheared into 200–1000 bp fragments by sonication for 2×8 min at 30 sec intervals using a Bioruptor UCD-200 (*Diagenode*) and diluted 1∶10 in ChIP dilution buffer (0.01% SDS, 1.1% Triton X-100, 1.2 mM EDTA, 16.7 mM Tris pH 8.1, 150 mM NaCl).

Immunoprecipitation was performed with the EZ ChIP Kit (*Millipore*). For each IP, 1×10^9^ ring stage parasites, 2.5×10^8^ trophozoites or 1×10^8^ schizonts were used. Optimal antibody dilutions were determined empirically and ranged between 1∶50 and 1∶200.

### Real time qPCR analysis

Primers amplifying upstream regions near the TSS (ups) and in the open reading frame (orf) were designed using the Primer Express software (*Applied Biosystems*) (Table S1). For genes whose TSS had not previously been experimentally determined and reported, predictions were obtained from the MAPP algorithm implemented at PlasmoDB [111]. Immunoprecipitated DNA and input DNA was quantified by real time qPCR (*Applied Biosystems* 7900HT) with SYBR PCR master mix (*Applied Biosystems*). Optimal PCR conditions were determined for each primer pair using serial dilutions of gDNA. PCR was performed in duplicates and melting curves were analysed after each run to confirm the specificity of the amplification. ChIP recoveries were normalized for input signals (ΔC_t_) and corrected for values obtained with non-immune control antibodies (ΔΔC_t_). The site specific enrichment of histones was calculated as the 2^−ΔΔCt^ value. To correct for differences in nucleosome density, enrichment of PfH2A.Z and H2A was expressed as a ratio over H3. As in the course of the study different H3 antibody lots were used to precipitate biological replicates, the H3 recoveries and therefore ratios varied considerably between experiments. However, the pattern of histone variant enrichment across genes was reproducible between experiments (Figure 3, 4 & S4).

For statistical analysis of PfH2A.Z enrichment in 3D7ΔSir2A and 3D7 parasites, the ratio of PfH2A.Z enrichment near the TSS and in the open reading frame (ups/orf ratio) was calculated. Paired t-test was performed using the GraphPad Prism software (Version 4).

### Mononucleosome co-immunoprecipitation

Mononucleosomes were prepared from freshly isolated nuclei by MNase (NEB) digestion with 20 KU MNase per 1×10^9^ IE and extraction with salt-free buffers [33]. Mononucleosomes from 2.5×10^8^ IE were incubated with 10 µl of antiserum overnight at 4°C and precipitated with 30 µl protein G agarose (*Millipore*). After extensive washing proteins were eluted with 2 x SDS PAGE loading buffer and analysed by SDS-PAGE and Western Blotting.

### Transcription analysis

Total RNA was harvested in parallel to the chromatin preparation by lysis of pelleted IEs in 20 pellet volumes of TRIzol (*Invitrogen*). RNA was purified as described previously [112] and cDNA was generated using Superscript III Reverse Transcriptase (*Invitrogen*). Quantitative RT-PCR was performed as described previously [47] using gene specific primers targeting the open reading frame, listed in Table S1. The level of each sequence in cDNA was determined relative to its level in a constant quantity of 3D7 strain gDNA and the amounts of cDNA and gDNA were normalised using the housekeeping gene arginyl-tRNA synthetase or the sbp1 gene by 2^−ΔΔCt^ analysis (Applied Biosystems user bulletin 2).

## Supporting Information

Figure S1 Multiple sequence alignment of deduced amino acid sequences of H2A.Z orthologues from different species. Identical and similar amino acids are shaded in black and grey, respectively. The region underlined in blue denotes the histone fold region, comprising alpha-helical elements (red) as well as loop 1 for H2A.Z/H2A.Z self interaction and loop 2 for nucleosome/DNA interaction (green). The region underlined in yellow encompasses the C-terminal docking domain which mediates interaction with the H3/H4 dimer. Lysine residues printed in red in PfH2A.Z were shown to be acetylated [60]. Blue letters represent critical amino acids for H2A.Z/H2A.Z interaction [61]. Alignments were performed with the BioEdit software (version 7).
(0.98 MB TIF)

Figure S2 Nuclear localization of PfH2A.ZGFP. Confocal microscopy of PfH2A.ZGFP parasites at the (A) merozoite, (B) ring, (C) trophozoite and (D) schizont stage. Blue: DNA stained with DAPI. Green: PfH2A.ZGFP. Overlays in the lower panels in each picture demonstrate the nuclear localization of PfH2A.ZGFP.
(1.27 MB TIF)

Figure S3 Biological replicate of ChIP analysis of PfH2A.Z occupancy in genes. ChIP was performed in ring (pale gray bars), trophozoite (mid gray bars) and schizont stage (black bars) parasites with antibodies against PfH2A.Z and H3 and non-immune control antibodies. Real time qPCR was performed targeting sequences near the TSS and in the open reading frame of genes with different expression profiles (constitutive, sporozoite, ring or schizont specific). Enrichment was calculated and the data are presented as ratio over H3 to correct for differences in nucleosome density in the inter- and intra-genic regions. The left scale corresponds to ring data, the right scale to trophozoite and schizont data. (A) Enrichment of PfH2A.Z/H3 in non-*var* genes. (B) Enrichment of H2A.Z/H3 in *var* genes *var2csa*, *var20* and *var41*. Amplified regions are depicted in the gene models under each graph. Error bars represent standard deviation from two technical replicates.
(0.40 MB TIF)

Figure S4 Verification of stage specific transcription profiles. Quantitative real time PCR was performed on cDNA prepared from ring (light grey), trophozoite (mid grey) and schizont (dark grey) stage parasites. The levels of each sequence in cDNA was determined relative to its levels in a constant quantity of 3D7 strain gDNA and the amount of cDNA normalised using the housekeeping gene arginyl-tRNA synthetase by 2^−ΔΔCt^ analysis. (A) Stage specific expression of ring, schizont, and constitutively expressed genes was verified. Sporozoite specific genes were not expressed in any stage. (B) *Var2csa* was expressed at high levels in ring stages but not in trophozoites and schizonts. Transcripts of two other *var* genes (*var20* and *var41*) were undetectable.
(0.26 MB TIF)

Figure S5 ChIP analysis of PfH2A.Z and histone modifications in upstream regions and open reading frames in (A) ring stage and (B) schizont stage 3D7 parasites. ChIP enrichment (over pre-immune serum) in the upstream region (ups) and open reading frame (orf) is shown for each antibody. PfH2A.Z (light grey), H3K4me3 (dark grey) and H3K9ac (white) concomitantly show a significant increase in the ups region in comparison to the orf. In contrast, H3K9me3 (black) is not significantly enriched. Shown is the median boxed with 25th and 75th percentile and minimum/maximum values as whiskers. A non-parametric Mann-Whitney test was performed and significant differences are indicated. 12 genes were analysed (N = 12) in two technical replicates of one experiment.
(0.24 MB TIF)

Figure S6 Correlation between PfH2A.Z and histone modifications in the upstream region of genes in (A) ring and (B) schizont stage 3D7 parasites. Enrichment of PfH2A.Z (X-axis) positively correlates with H3K4me3 (p<0.0001) and H3K9ac (p<0.0001) and negatively correlates with H3K9me3 (p<0.0001) at both stages. P-value and spearman correlation coefficient (r) are indicated. A total of 36 genes were analysed, including 20 *var* genes. The *var* genes cluster together in a group with low PfH2A.Z enrichment and low euchromatic histone marks (H3K9ac and H3K4me3) but high levels of the heterochromatin mark H3K9me3. Data are compiled from two technical replicates of one ChIP experiment.
(0.40 MB TIF)

Figure S7 PfH2A.Z is enriched in the upstream region of a *var* gene dominantly transcribed in ICAM-selected 3D7 parasites. (A) qPCR analysis of ICAM selected parasites identifies PFL0020w as the most abundantly transcribed *var* gene. (B) Northern Blot analysis confirms PFL0020w as the dominant transcript in ICAM selected parasites. A PFL0020w specific probe labels a band of about 10 kb in ICAM selected rings stage parasites (3D7-ICAM), but not in unselected parasites (3D7). A probe representing the conserved exon 2 hybridizes to multiple transcripts in 3D7, but only one dominant transcript in 3D7-ICAM which corresponds to PFL0020w. (C) Model of the PFL0020w gene. The predicted transcription start site (TSS) at approximately −400 bp upstream of the start codon is represented by the arrow. Coordinates of loci amplified by qPCR are indicated below the graph. (D) ChIP analysis of PfH2A.Z distribution along the PFL0020w gene in 3D7-ICAM at ring stage (light grey bars), schizont stage (mid grey bars) or in unselected 3D7 ring stage parasites (dark grey bars). PfH2A.Z is enriched near the TSS at −400 bp at ring stage of 3D7-ICAM but not unselected parasites, and not in 3D7-ICAM schizonts. PfH2A.Z is also enriched in the intron of PFL0020w in both ICAM selected and unselected lines and slightly enriched in the sequence amplified by qPCR from DBL5 which is directly adjacent to the intron. Error bars represent two biological replicates.
(0.89 MB TIF)

Figure S8 Transcription profiles of *var* genes in 3D7ΔSir2A, 3D7ΔSir2B and 3D7 parasites at ring stage. Multiple *var* genes are expressed at high levels in 3D7ΔSir2A parasites (red bars) and 3D7ΔSir2B parasites (blue bars). Only marginal *var* gene expression is detected in 3D7 (black bars). Levels of each *var* gene sequence in cDNA were normalised using the gene arginyl-tRNA synthetase and expressed relative to the level of the same *var* gene sequence detected in a constant amount of 3D7 strain gDNA using 2^−ΔΔCt^ analysis. *Var* groups according to [113] are indicated above the graph and separated in boxes.
(0.39 MB TIF)

Figure S9 Correlation between PfH2A.Z and histone modifications in the upstream region of *var* genes in 3D7ΔSir2A trophozoites. Enrichment of PfH2A.Z (X-axis) positively correlates with H3K4me3 (upper panel) and negatively correlates with H3K9me3 (lower panel). P-value and spearman correlation coefficient (r) are indicated. 11 *var* genes were analysed in one experiment.
(0.09 MB TIF)

Table S1 Gene accession numbers and oligonucleotide sequences used for qPCR.
(0.08 MB PDF)

Video S1 PfH2A.Z shows a polarized distribution in the nucleus. 3D reconstruction using serial Z-stacks of confocal images was performed. The animation shows a 360° rotation. The blue area depicts DAPI-stained DNA, the green area depicts PfH2A.Z labelled with rabbit anti-PfH2A.Z antiserum. PfH2A.Z labelling is concentrated towards one side of the nucleus.
(0.04 MB MP4)
