# Supplementary figures and images for: Expression of P. falciparum var Genes Involves Exchange of the Histone Variant H2A.Z at the Promoter
Source: PLoS Pathog. 2011 Feb 17;7(2):e1001292. doi: 10.1371/journal.ppat.1001292 (PMC3040674; doi:10.1371/journal.ppat.1001292)

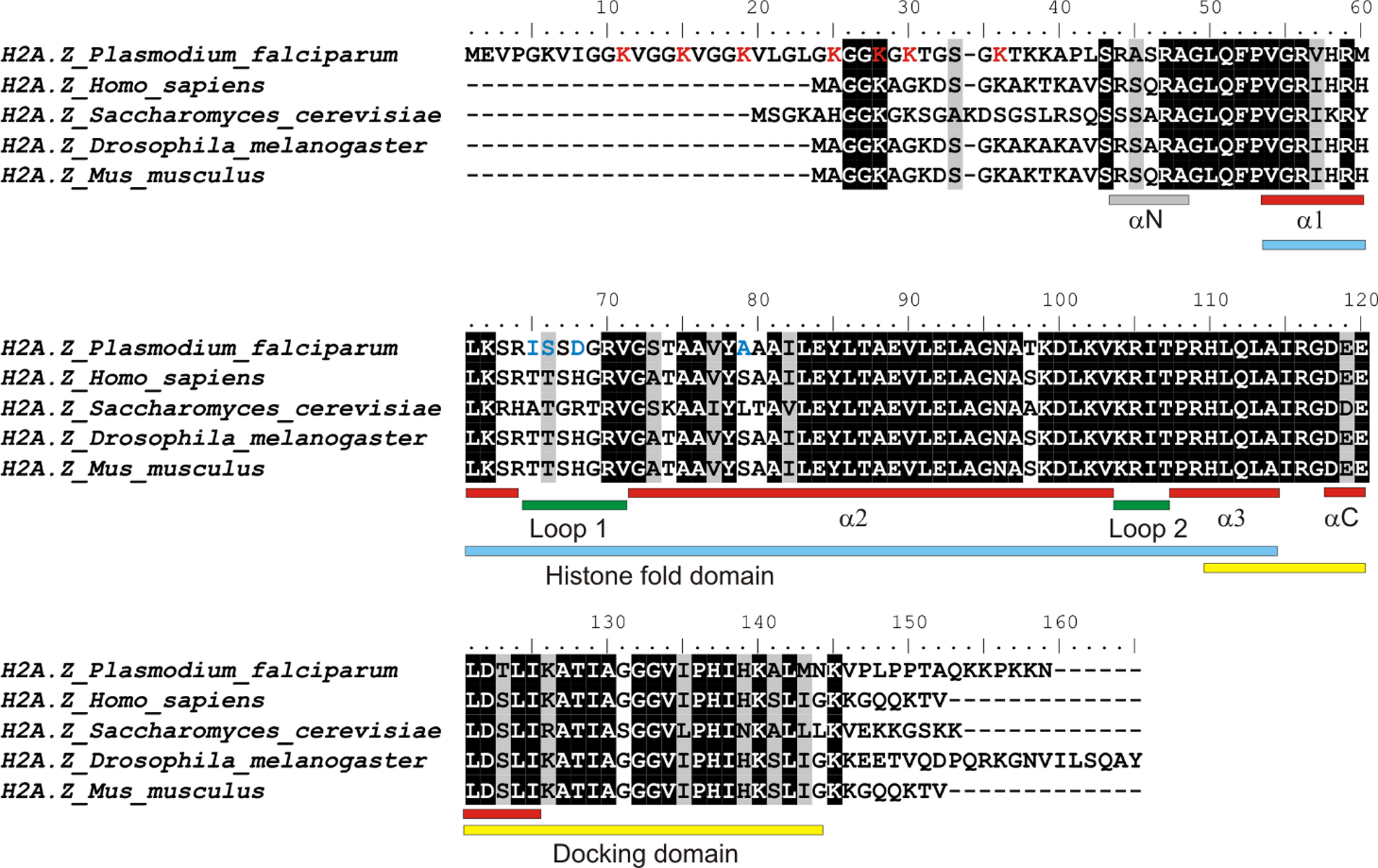

Supplement: Figure S1 — Multiple sequence alignment of deduced amino acid sequences of H2A.Z orthologues from different species. Identical and similar amino acids are shaded in black and grey, respectively. The region underlined in blue denotes the histone fold region, comprising alpha-helical elements (red) as well as loop 1 for H2A.Z/H2A.Z self interaction and loop 2 for nucleosome/DNA interaction (green). The region underlined in yellow encompasses the C-terminal docking domain which mediates interaction with the H3/H4 dimer. Lysine residues printed in red in PfH2A.Z were shown to be acetylated [60]. Blue letters represent critical amino acids for H2A.Z/H2A.Z interaction [61]. Alignments were performed with the BioEdit software (version 7). (0.98 MB TIF) [file ppat.1001292.s001.tif]

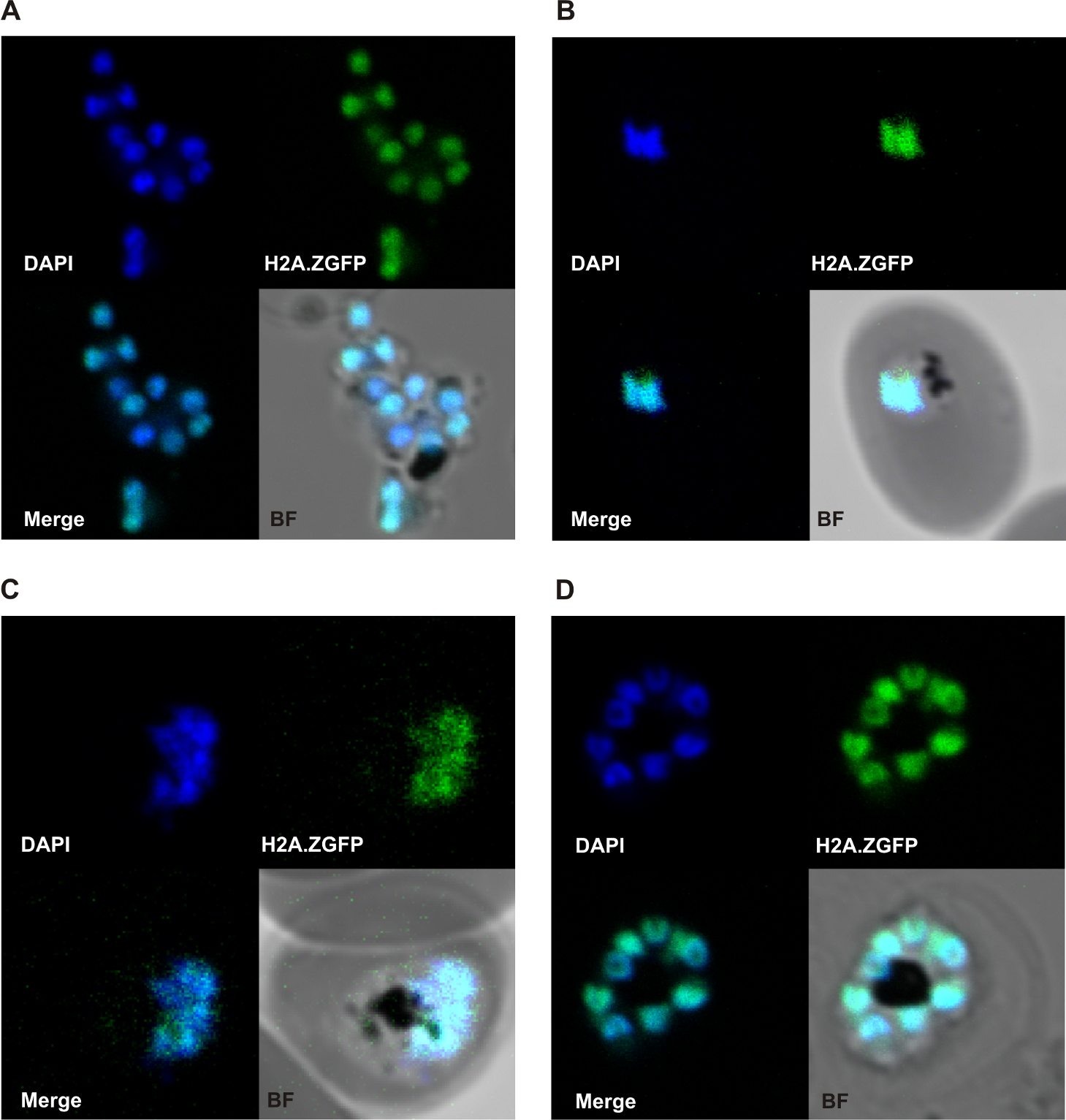

Supplement: Figure S2 — Nuclear localization of PfH2A.ZGFP. Confocal microscopy of PfH2A.ZGFP parasites at the (A) merozoite, (B) ring, (C) trophozoite and (D) schizont stage. Blue: DNA stained with DAPI. Green: PfH2A.ZGFP. Overlays in the lower panels in each picture demonstrate the nuclear localization of PfH2A.ZGFP. (1.27 MB TIF) [file ppat.1001292.s002.tif]

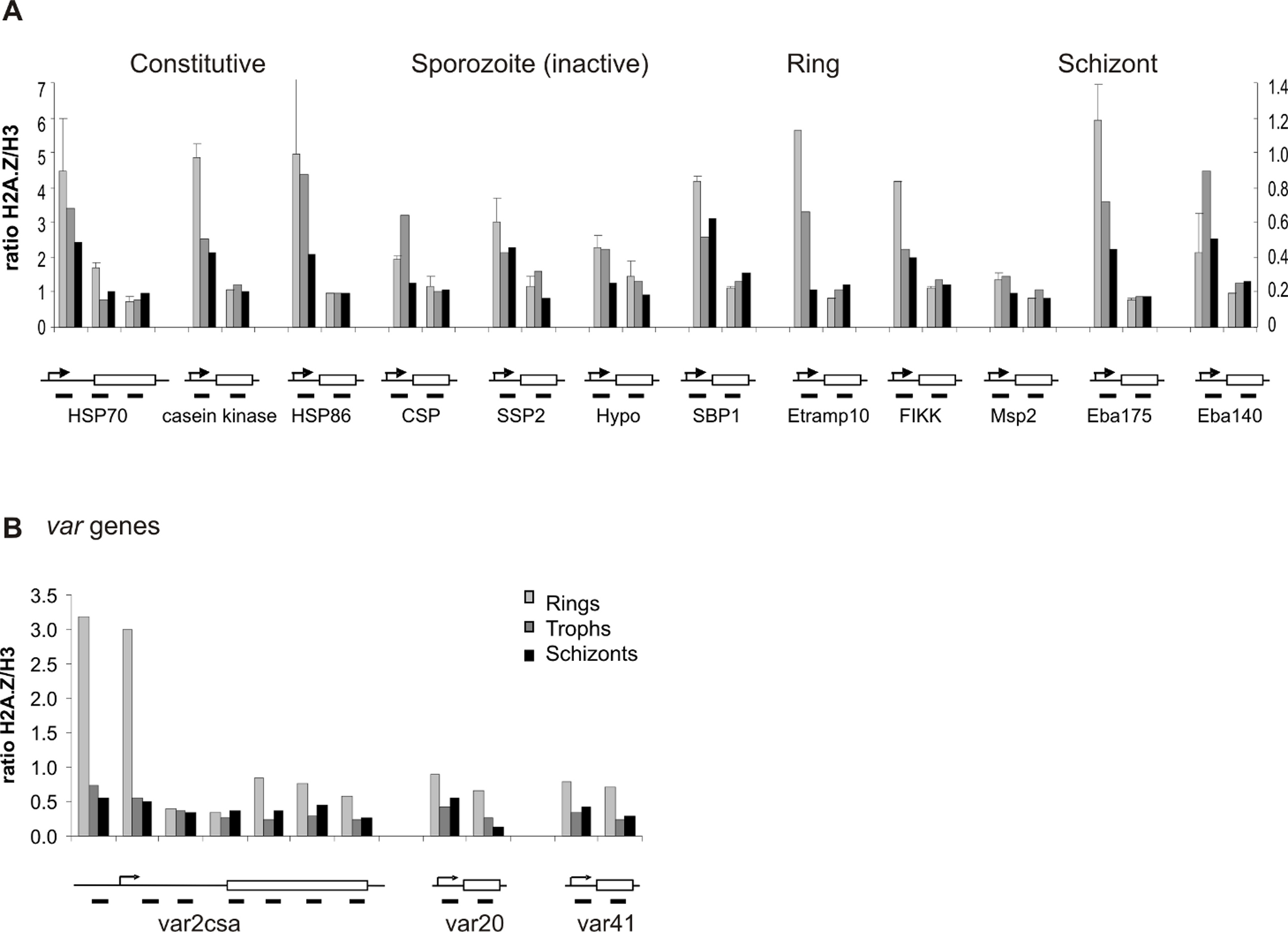

Supplement: Figure S3 — Biological replicate of ChIP analysis of PfH2A.Z occupancy in genes. ChIP was performed in ring (pale gray bars), trophozoite (mid gray bars) and schizont stage (black bars) parasites with antibodies against PfH2A.Z and H3 and non-immune control antibodies. Real time qPCR was performed targeting sequences near the TSS and in the open reading frame of genes with different expression profiles (constitutive, sporozoite, ring or schizont specific). Enrichment was calculated and the data are presented as ratio over H3 to correct for differences in nucleosome density in the inter- and intra-genic regions. The left scale corresponds to ring data, the right scale to trophozoite and schizont data. (A) Enrichment of PfH2A.Z/H3 in non-var genes. (B) Enrichment of H2A.Z/H3 in var genes var2csa, var20 and var41. Amplified regions are depicted in the gene models under each graph. Error bars represent standard deviation from two technical replicates. (0.40 MB TIF) [file ppat.1001292.s003.tif]

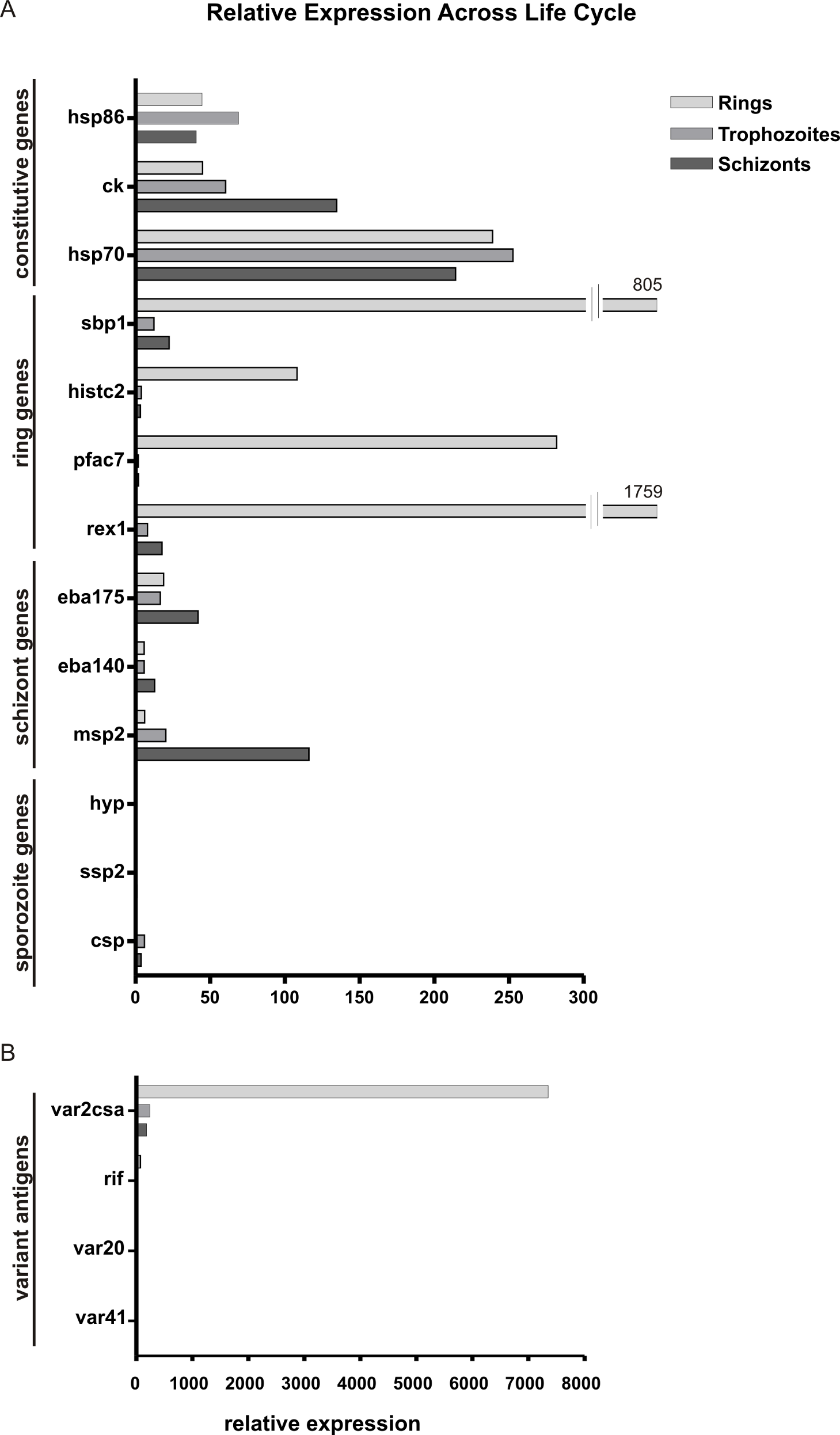

Supplement: Figure S4 — Verification of stage specific transcription profiles. Quantitative real time PCR was performed on cDNA prepared from ring (light grey), trophozoite (mid grey) and schizont (dark grey) stage parasites. The levels of each sequence in cDNA was determined relative to its levels in a constant quantity of 3D7 strain gDNA and the amount of cDNA normalised using the housekeeping gene arginyl-tRNA synthetase by 2−ΔΔCt analysis. (A) Stage specific expression of ring, schizont, and constitutively expressed genes was verified. Sporozoite specific genes were not expressed in any stage. (B) Var2csa was expressed at high levels in ring stages but not in trophozoites and schizonts. Transcripts of two other var genes (var20 and var41) were undetectable. (0.26 MB TIF) [file ppat.1001292.s004.tif]

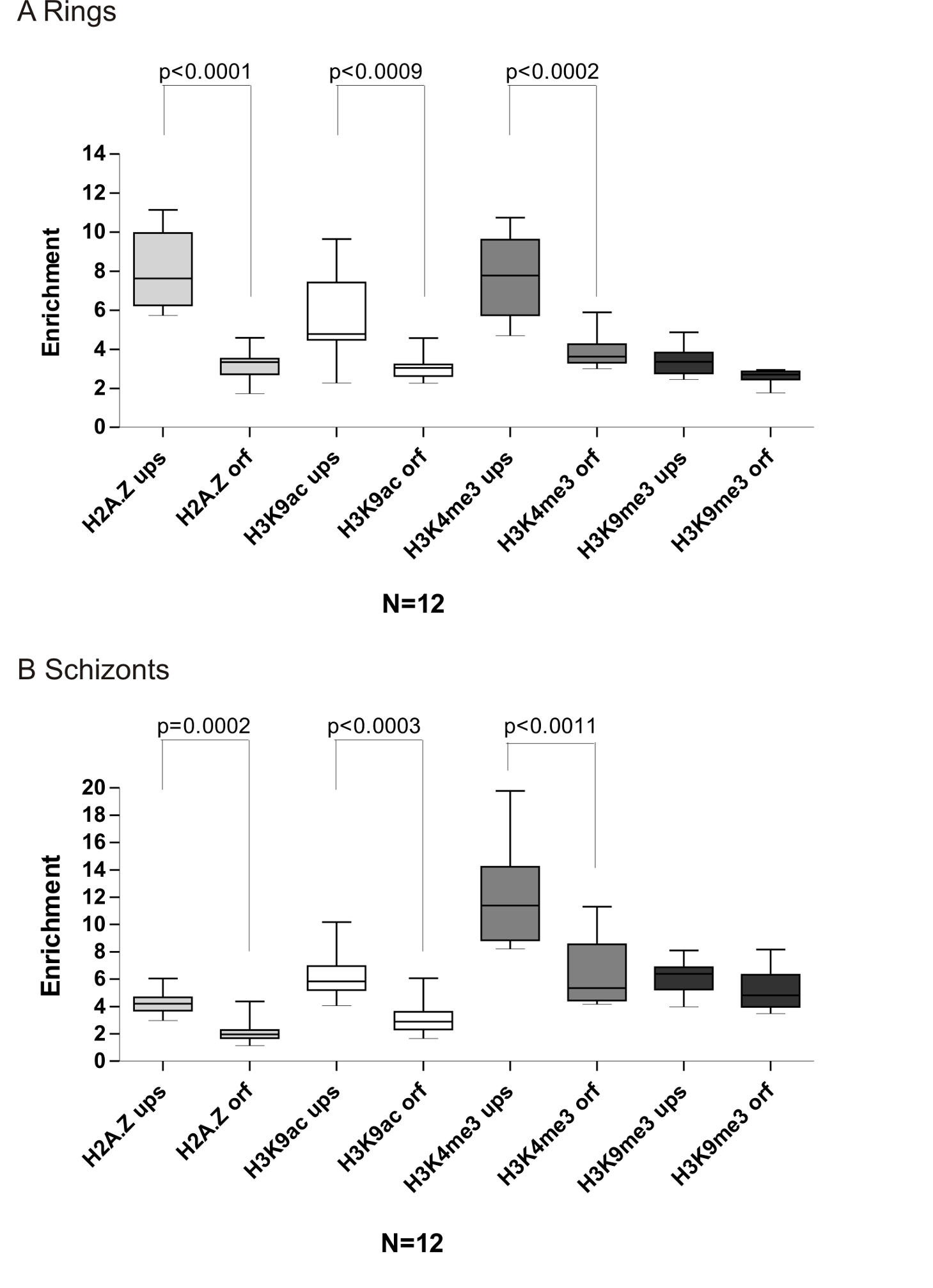

Supplement: Figure S5 — ChIP analysis of PfH2A.Z and histone modifications in upstream regions and open reading frames in (A) ring stage and (B) schizont stage 3D7 parasites. ChIP enrichment (over pre-immune serum) in the upstream region (ups) and open reading frame (orf) is shown for each antibody. PfH2A.Z (light grey), H3K4me3 (dark grey) and H3K9ac (white) concomitantly show a significant increase in the ups region in comparison to the orf. In contrast, H3K9me3 (black) is not significantly enriched. Shown is the median boxed with 25th and 75th percentile and minimum/maximum values as whiskers. A non-parametric Mann-Whitney test was performed and significant differences are indicated. 12 genes were analysed (N = 12) in two technical replicates of one experiment. (0.24 MB TIF) [file ppat.1001292.s005.tif]

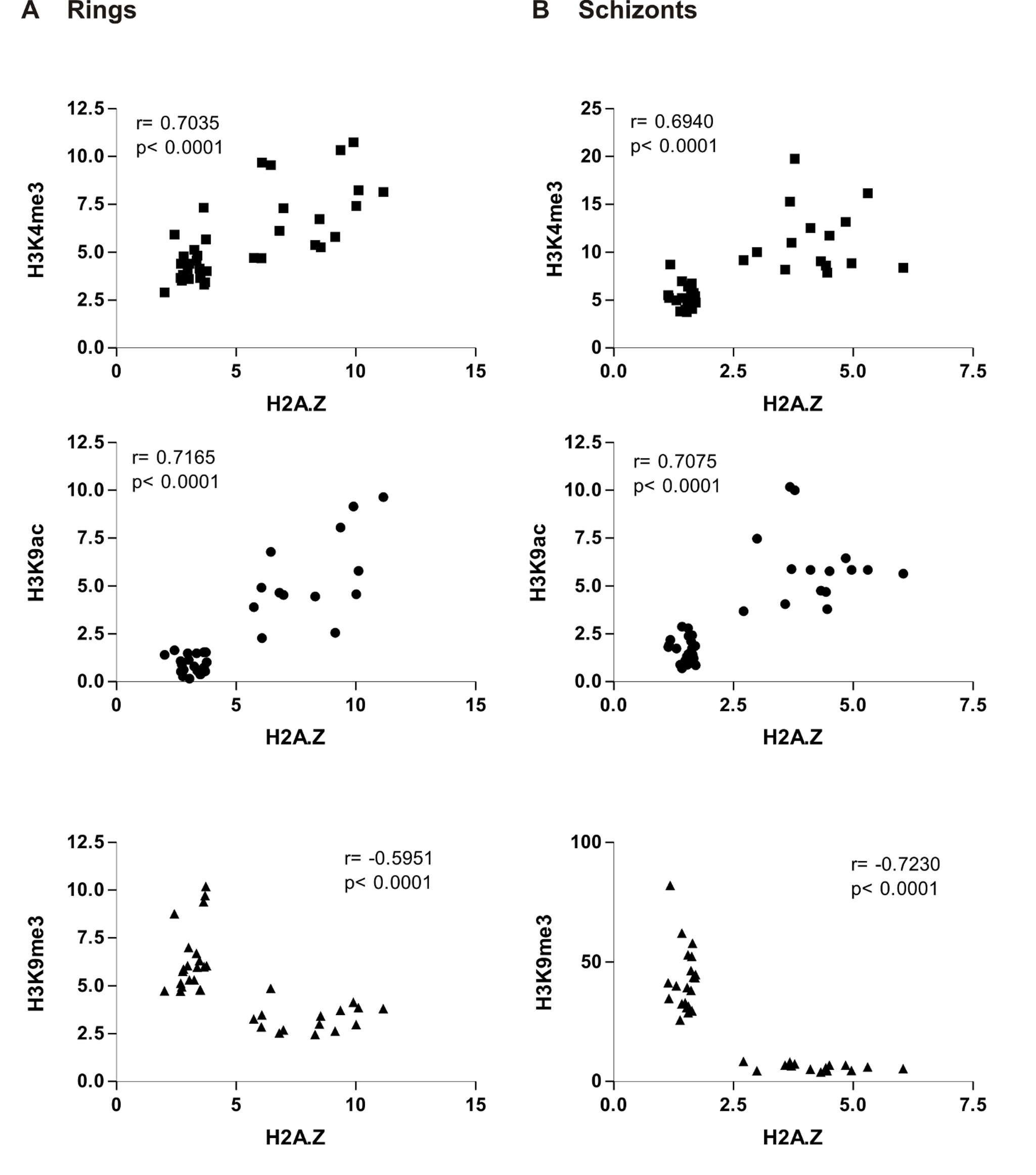

Supplement: Figure S6 — Correlation between PfH2A.Z and histone modifications in the upstream region of genes in (A) ring and (B) schizont stage 3D7 parasites. Enrichment of PfH2A.Z (X-axis) positively correlates with H3K4me3 (p<0.0001) and H3K9ac (p<0.0001) and negatively correlates with H3K9me3 (p<0.0001) at both stages. P-value and spearman correlation coefficient (r) are indicated. A total of 36 genes were analysed, including 20 var genes. The var genes cluster together in a group with low PfH2A.Z enrichment and low euchromatic histone marks (H3K9ac and H3K4me3) but high levels of the heterochromatin mark H3K9me3. Data are compiled from two technical replicates of one ChIP experiment. (0.40 MB TIF) [file ppat.1001292.s006.tif]

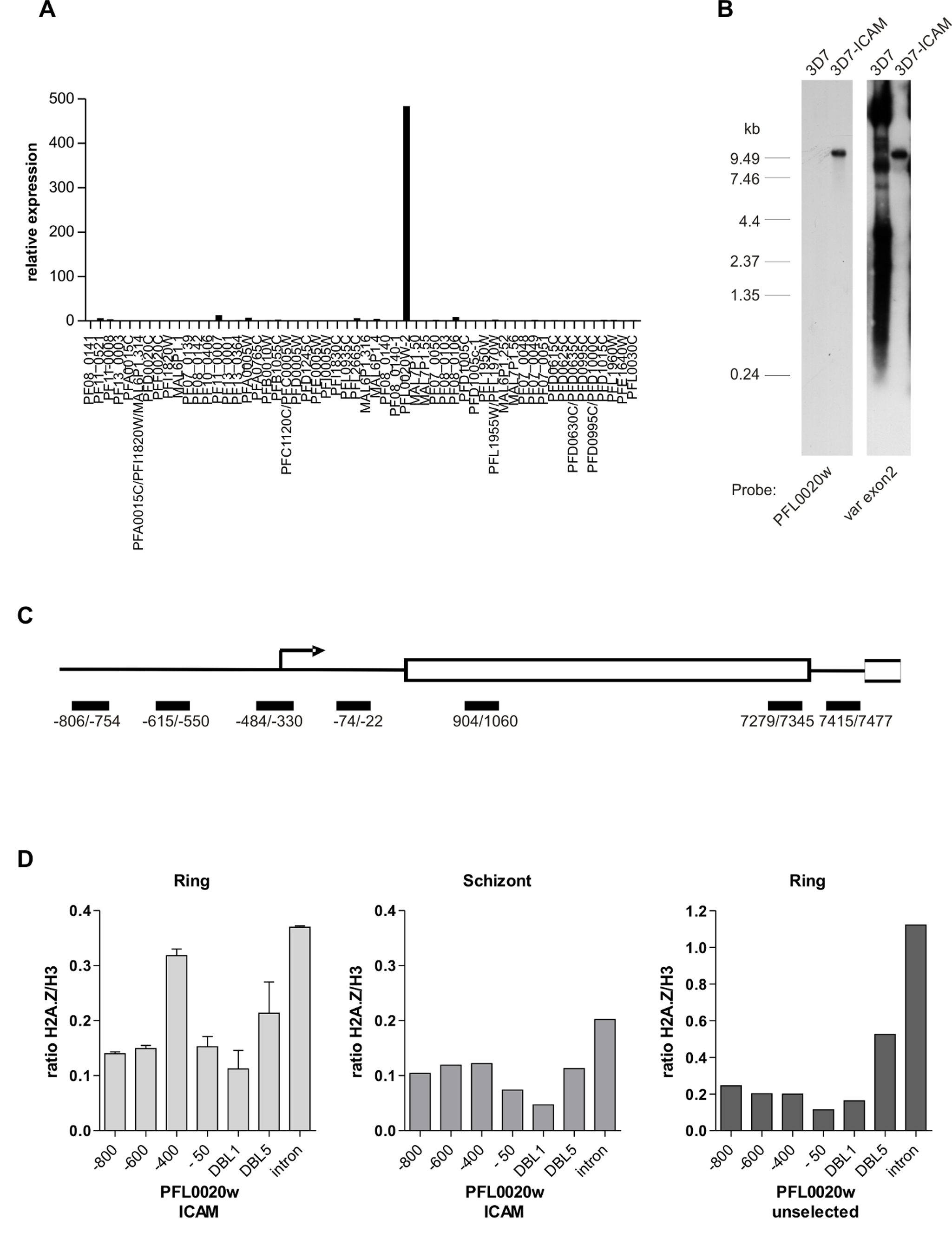

Supplement: Figure S7 — PfH2A.Z is enriched in the upstream region of a var gene dominantly transcribed in ICAM-selected 3D7 parasites. (A) qPCR analysis of ICAM selected parasites identifies PFL0020w as the most abundantly transcribed var gene. (B) Northern Blot analysis confirms PFL0020w as the dominant transcript in ICAM selected parasites. A PFL0020w specific probe labels a band of about 10 kb in ICAM selected rings stage parasites (3D7-ICAM), but not in unselected parasites (3D7). A probe representing the conserved exon 2 hybridizes to multiple transcripts in 3D7, but only one dominant transcript in 3D7-ICAM which corresponds to PFL0020w. (C) Model of the PFL0020w gene. The predicted transcription start site (TSS) at approximately −400 bp upstream of the start codon is represented by the arrow. Coordinates of loci amplified by qPCR are indicated below the graph. (D) ChIP analysis of PfH2A.Z distribution along the PFL0020w gene in 3D7-ICAM at ring stage (light grey bars), schizont stage (mid grey bars) or in unselected 3D7 ring stage parasites (dark grey bars). PfH2A.Z is enriched near the TSS at −400 bp at ring stage of 3D7-ICAM but not unselected parasites, and not in 3D7-ICAM schizonts. PfH2A.Z is also enriched in the intron of PFL0020w in both ICAM selected and unselected lines and slightly enriched in the sequence amplified by qPCR from DBL5 which is directly adjacent to the intron. Error bars represent two biological replicates. (0.89 MB TIF) [file ppat.1001292.s007.tif]

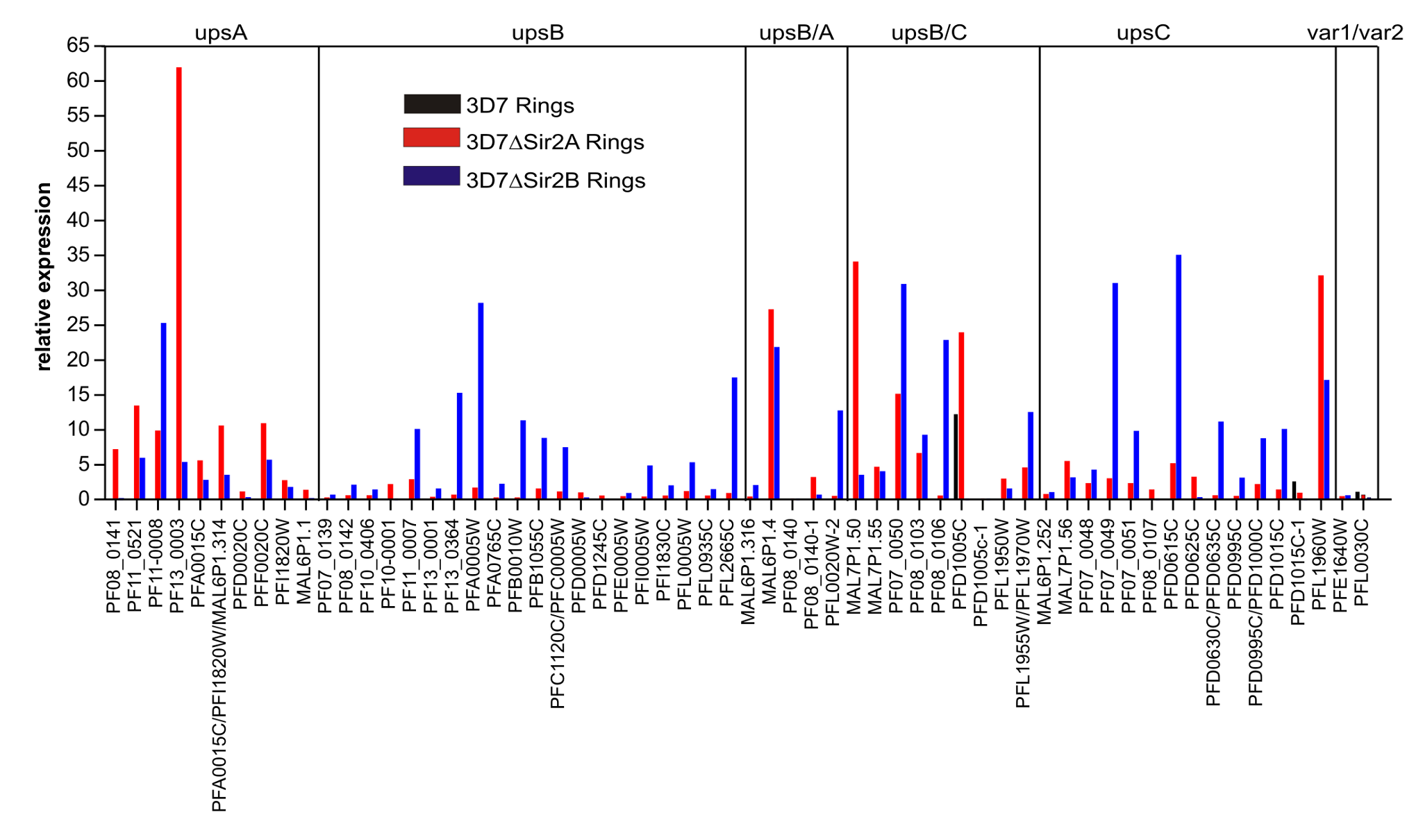

Supplement: Figure S8 — Transcription profiles of var genes in 3D7ΔSir2A, 3D7ΔSir2B and 3D7 parasites at ring stage. Multiple var genes are expressed at high levels in 3D7ΔSir2A parasites (red bars) and 3D7ΔSir2B parasites (blue bars). Only marginal var gene expression is detected in 3D7 (black bars). Levels of each var gene sequence in cDNA were normalised using the gene arginyl-tRNA synthetase and expressed relative to the level of the same var gene sequence detected in a constant amount of 3D7 strain gDNA using 2−ΔΔCt analysis. Var groups according to [113] are indicated above the graph and separated in boxes. (0.39 MB TIF) [file ppat.1001292.s008.tif]

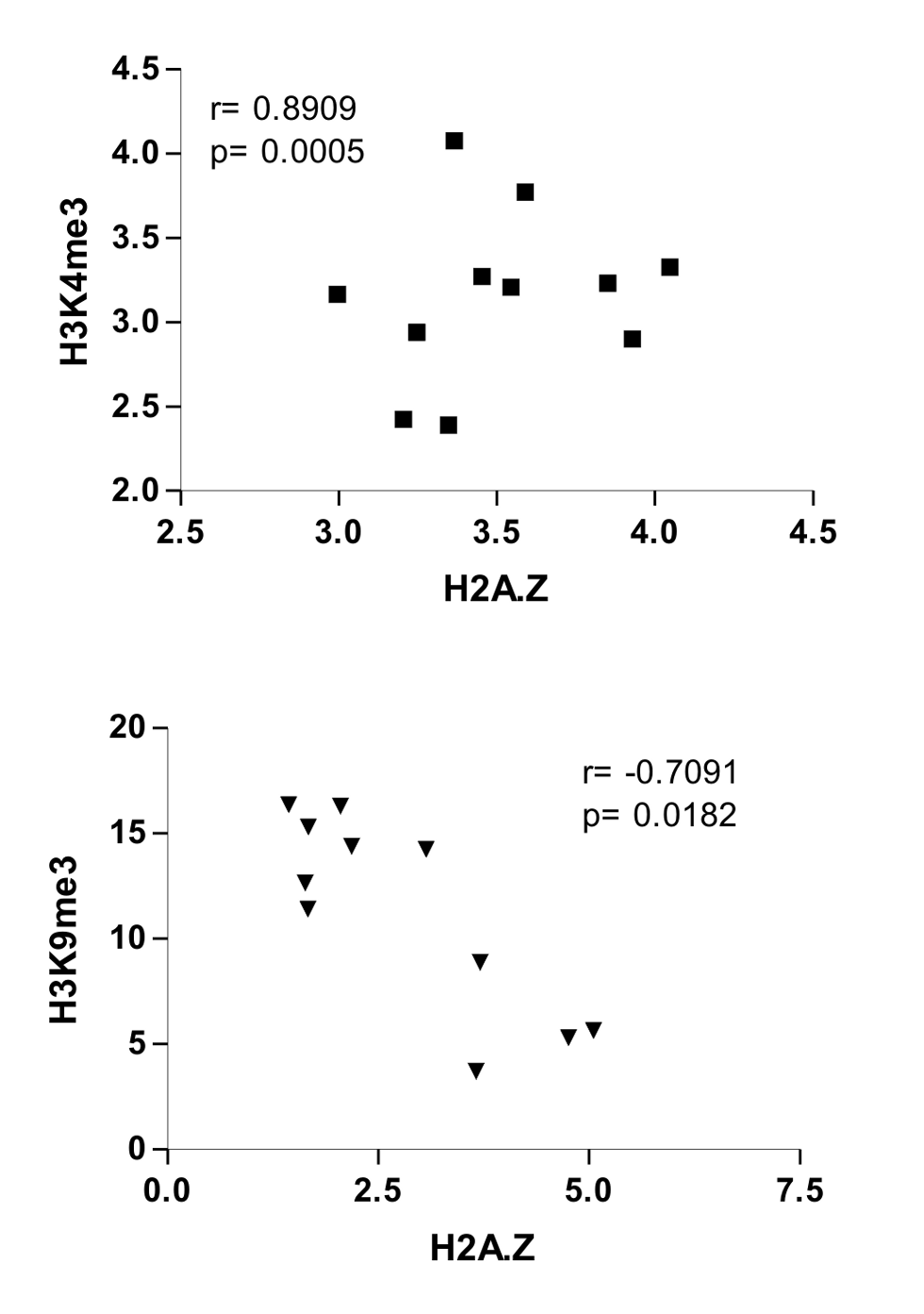

Supplement: Figure S9 — Correlation between PfH2A.Z and histone modifications in the upstream region of var genes in 3D7ΔSir2A trophozoites. Enrichment of PfH2A.Z (X-axis) positively correlates with H3K4me3 (upper panel) and negatively correlates with H3K9me3 (lower panel). P-value and spearman correlation coefficient (r) are indicated. 11 var genes were analysed in one experiment. (0.09 MB TIF) [file ppat.1001292.s009.tif]
