# Supplementary material for: Expression of P. falciparum var Genes Involves Exchange of the Histone Variant H2A.Z at the Promoter
Source: PLoS Pathog. 2011 Feb 17;7(2):e1001292. doi: 10.1371/journal.ppat.1001292 (PMC3040674; doi:10.1371/journal.ppat.1001292)
Supplement: Table S1 — Gene accession numbers and oligonucleotide sequences used for qPCR. (0.08 MB PDF) [file ppat.1001292.s010.pdf]

Table S1. Gene accession numbers and oligonucleotide sequences used for qPCR

Oligo	Target	Sequence	Reference	Stage
**Stage specific genes**				
**125**	PF07_0029 hsp86 ups For	GCCATTGGATATATATTTAGTATTCCA		constitutive
**126**	PF07_0029 hsp86 ups Rev	TCTTTAAATTCATGCAAAAATTTACTATAA		
**127**	PF07_0029 hsp86 (+) 1434 For	CACCGAATTACTCCGATTCCA		
**128**	PF07_0029 hsp86 (+) 1434 Rev	TCCGATCATTTCGTCTCCTGA		
**27**	PF08_0054 hsp70 (-) 1127 For	AAACAGAAGGGGCGAAATGG	[1]	constitutive
**28**	PF08_0054 hsp70 (-) 1127 Rev	GCAAGTTCAAGGGACTAAAATTCG	[1]	
**29**	PF08_0054 hsp70 (+) 3 For	GGCTAGTGCAAAAGGTTCAAAACC	[1]	
**30**	PF08_0054 hsp70 (+) 3 Rev	CGGTATCGGTGAAAGCAACATAAG	[1]	
**31**	PF08_0054 hsp70 (+)1425 For	GGTATTCCACCTGCACCAAGAA	[1]	
**32**	PF08_0054 hsp70 (+)1425 Rev	CAGCCGTAACGTTTAAGATACCGT	[1]	
**37**	PF11_0377 casein kinase I (-)1782 For	TATATTCATGTCATGCACTCTTCCC		constitutive
**38**	PF11_0377 casein kinase I (-)1782 Rev	TTAAGTCAGGCGTTCTTAGAAATCTTT		
**39**	PF11_0377 casein kinase I (+)636 For	TGGTGTACCTAAAGTATATTGGTACGGT		
**40**	PF11_0377 casein kinase I (+)636 Rev	AAGGGATGGGCCTAATAAATCAA		
**41**	PFC0210c csp (-)636 For	GAGGGGTAAAGGGGGGCTTAA	[1]	Sporozoite
**42**	PFC0210c csp (-)636 Rev	AACATTATATGCTTCCTTAAGAACATATAGTGAAT	[1]	
**43**	PFC0210c csp (+)211 For	TCACTTGGAGAAAATGATGATGGA	[1]	
**44**	PFC0210c csp (+)211 Rev	CCATCCGCTGGTTGCTTTA	[1]	
**45**	PF13_0201 ssp2 TRAP (-)938 For	TTTTTACATGTATAGTGAATGAGGTGTCCT	[1]	Sporozoite
**46**	PF13_0201 ssp2 TRAP (-)938 Rev	TCAGATTTATTCAAACGCATGCTG	[1]	
**47**	PF13_0201 ssp2 TRAP (+)148 For	CCTTCTAATGGATTGTTCTGGAAGT	[1]	
**48**	PF13_0201 ssp2 TRAP (+)148 Rev	TTTCATAGCTAGAGGTACTGCATGG	[1]	
**49**	PF11_0480 hypothetical (-)224 For	ATTGTGTTACATATTTTAGTTGACAACCTTT		Sporozoite
**50**	PF11_0480 hypothetical (-)224 Rev	TGAATGTCAATTTTTGGATAGGACA		
**51**	PF11_0480 hypothetical (+)8259 For	TTCAGTTGGTTTTAGTAACAGAAGCAA		
**52**	PF11_0480 hypothetical (+)8259 Rev	TAAGAAGGGCAAATTGTCCATCTATA		
**123**	PFB0300c msp2 (-)1234 For	GATCAAAATAGAAAATCATGTGCCATA		Schizont
**124**	PFB0300c msp2 (-)1234 Rev	TGATCATCAGAACATCCACCATTAT		
**25**	PFB0300c msp2 (+)538 For	TCCTGTACCTTTATTCTCTGGTG	[1]	
**26**	PFB0300c msp2 (+)538 Rev	CAAGCTGAAAATTCTGCTCC	[1]	
**57**	MAL7P1.176 Eba-175 (ups) For	GATACAATTTGAATTACGTCATTCCA		Schizont
**58**	MAL7P1.176 Eba-175 (ups) Rev	TCTTATTTATTACTTTATTTGGTTTTCCTTGT		

**59**	MAL7P1.176 Eba-175 (coding) For	GAACGGAAACTCGTACGGATGA	
**60**	MAL7P1.176 Eba-175 (coding) Rev	CTCTCCTACACTTTGTTGTGGATTCT	
**53**	MAL13P1.60 Eba140 (ups) For	ATATCATACATTTTGGAAATGGAAA	Schizont
**54**	MAL13P1.60 Eba140 (ups) Rev	ATGCTTAACATAATGCGACTGAG	
**55**	MAL13P.160 Eba140 (coding) For	CCAGAAGACAAACTTTATGTCTTGGA	
**56**	MAL13P.160 Eba140 (coding) Rev	TGACCACGATGTAAAAGATATGTACG	
**61**	PFI1735c Rex (-392) For	AATATTATAAAATATTAAAATTTTCTTGTATTCCT	Ring
**62**	PFI1735c Rex (-392) Rev	ACATATATATATAATATGTAAAAGAACGTGCA	
**63**	PFI1735c Rex (coding) For	AAGATATGCAAAGCCCATTTGATT	
**64**	PFI1735c Rex (coding) Rev	GGGAGCAAAGATTGTGTACTTACGA	
**94**	PFE0065c SBP1 ups (-)1308 For	GTATTATTATTATATACGTATCAATGTATGATC	Ring
**95**	PFE0065c SBP1 ups Rev	ATGTAACATATGTATCATTATAAAGGGA	
**MD93**	PFE0065c SBP1 orf For	TTAGCCGACGAACCAACACA [2]	
**MD94**	PFE0065c SBP1 orf Rev	TTCGGTTGTCTCTGGTACTGCA [2]	
**156**	PFL0040c FIKK ups For	TGAGAAAAGCAAATTGAGTACTTTTAA	Ring
**157**	PFL0040c FIKK ups Rev	ATCTGGGGGTCCTACGATATAT	
**154**	PFL0040c FIKK orf For	GAATCATGCGTACCAACTATAGGA	
**155**	PFL0040c FIKK orf Rev	CTATTGGTGTATATTCACGTTTTCC	
**160**	PFL0035c AC7 ups For	AATTTTCTGTACATTTTTAACGCTCATT	Ring
**161**	PFL0035c AC7 ups Rev	TCCCTCTATAAGAGGAAATATAAACAAA	
**KB6**	PFL0035c AC7 orf For	GTGCGGAAAAGCCTATTGGA	
**KB7**	PFL0035c AC7 orf Rev	CACTATATGGTAATGGTCTTTGTATTTGTTT	
**198**	hist c 2 ups -777 For	AAATATTTCGGTTTTTCTGGATTAAT	Ring
**199**	hist c 2 ups -777 Rev	TTTTTCCTACAGTTTCCTTTATCATATA	
**200**	hist c 2 orf +1640 For	CCTCATCGGTGTCAAATTCAA	
**201**	hist c 2 orf +1640 Rev	TCCGAAGCTCCTAAGTAATTTTCA	
***Var* genes**			
**131**	var2CSA (-1500) For	TGCTTCATAAATAAAACATGCAATATAA	
**132**	var2CSA (-1500) Rev	TCTAAAATACCTCTCTTAACTCTATAACCAGT	
**129**	var2CSA (-1000) For	TATTTCTTTTTATACAATAATGCATGGT	
**130**	var2CSA (-1000) Rev	TGATTTGTTTGTATATTTTTAGGTTAATTT	
**71**	var2CSA (-575) For	TGTGAATGCAATGACAGAAT	
**72**	var2CSA (-575) Rev	TCCTTACAGGTTCATAAGTGC	
**75**	var2CSA coding (+75) For	ATCGTTGAAAGCTGATCCTA	
**76**	var2CSA coding (+75) Rev	ATTTCCATTGGTCATTATCG	

**MD191**	var2CSA DBL1 For	CAAAAATACGAGAAAACGACAAGGT	[2]	
**MD192**	var2CSA DBL1 Rev	GGTCCTTTGGGTATTTATCTTGAAG	[2]	
**MD64**	var2CSA DBL3 For	TGTCATGCTGTTCAAAGAAGTTTTATT	[2]	
**MD65**	var2CSA DBL3 Rev	TGGTACCCAAAATCATATTCTTATAATCA	[2]	
**77**	var2CSA DBL6 For	TGTGGATACAAACATGCCTA		
**78**	var2CSA DBL6 Rev	CAGTCCATTCTTGAAACCAT		
**192**	var2CSA intron For	TACATGCATATATATGCACAAAATTTA		
**193**	var2CSA intron Rev	TGTATTTAGGTAGGTATGTATGTATGTGA		
**158**	PFL0020w var -50 For	ACAATACTCCCATAACATACGCAATAC		
**159**	PFL0020w var -50 Rev	GGTTCGTGTGGGGAGTGG		
**AS9**	PFL0020w var DBL1 For	TCGATTATGTGCCGCAGTAT	[3]	
**AS9**	PFL0020w var DBL1 Rev	TTCCCGTACAATCGTATCCA	[3]	
**202**	PFL0020w intron For	ATGTATGTTTGAGCGTTTGGATA		
**203**	PFL0020w intron Rev	ACATATACAGAAACACATATACGTCCA		
**196**	PF08_0141 ups For	TGTTGTAGGTGATGAATGTATGGA		
**197**	PF08_0141 ups Rev	TAATGTTAATAAATCCTCATATTCATGTAA		
**AS41**	PF08_0141 orf For	GGTGTCAAGGCAGCTAATGA	[3]	
**AS41**	PF08_0141 orf Rev	TATGTCCTGCGCTATTTTGC	[3]	
**194**	PF08_0141 intron For	TACATGCATATATATGCACAAAATTTAT		
**195**	PF08_0141 intron Rev	AGGTACGTATATGTGTATGGGTATGTA		
**170**	MAL7P1.50 ups For	CGATACGTTCTTGTTCTCGATT		
**171**	MAL7P1.50 ups Rev	TGACATATCCTGTAGTATAACGTAGAAAT		
**MF4F**	MAL7P1.50 orf For	GGTGGAGGTAGTCCACAGGA	[4]	
**MF4R**	MAL7P1.50 orf Rev	CAGCTATTTCCCCACCAGAA	[4]	
**172**	PFD1005c ups For	AAAGGAACAAGTTTTTTTCAAACTA		
**173**	PFD1005c ups Rev	AAGATCGAGAACAAAAGGGTATC		
**RD2F**	PFD1005c orf For	ACGATTGGTGGGAAACAAAT	[5]	
**RD2R**	PFD1005c orf Rev	CCCCATTCTTTTATCCATCG	[5]	
**174**	MAL6P1.316 (PFF0010w) ups For	TTTGTGACAATGGAGAAAATAGAA		
**175**	MAL6P1.316 (PFF0010w) ups Rev	TTTGCAATCTTGATAACATAATGTTT		
**AS94**	MAL6P1.316 (PFF0010w) orf For	TGGAAAGAACATGGACCTGA	[3]	
**AS94**	MAL6P1.316 (PFF0010w) orf Rev	TTCCTCGAGGGAAGAATCAC	[3]	
**176**	PFD0020c ups For	ATATTATTCTATTTGTATACGTGATGACATAA		
**177**	PFD0020c ups Rev	TGACATTGGAGAAAATAGAACAAATT		
**AS25**	PFD0020c orf For	ATATGGGAAGGGATGCTCTG	[3]	
**AS25**	PFD0020c orf Rev	TGAACCATCGAAGGAATTGA	[3]	

**178**	PFD0995c/PFD1000c ups For	GCCACAATCACGGCATCA		
**179**	PFD0995c/PFD1000c ups Rev	AACAAACAATAAGGAAGATATCAACGAA		
**AS95**	PFD0995c/PFD1000c orf For	TCACAACCTGACCCCCTACT	[3]	
**AS95**	PFD0995c/PFD1000c orf Rev	TCTTCGTCGTTGTCATCCTC	[3]	
**180**	PF08_0106 ups For	CCATAACATACATACAATCACCCC		
**181**	PF08_0106 ups Rev	GCATGGTTCTTGAAGTGGTAG		
**AS55**	PF08_0106 orf For	TTTGTCCGGAAGACGATACA	[3]	
**AS55**	PF08_0106 orf Rev	ATCTGGGGCAGAATTACCAC	[3]	
**148**	PF07_0050 ups For	AAATATGATAGATAATATAGATAGAGAGAAACG		
**149**	PF07_0050 ups Rev	CAAAAGAGACAAATATGTTCTTCGT		
**AS46**	PF07_0050 orf For	GCGACGCTCAAAAACATTTA	[3]	
**AS46**	PF07_0050 orf Rev	TCATCCAACGCAATCTTTGT	[3]	
**162**	PF13_0003 ups For	TATATAATGGAAAATCTTTGGTATAGAAAA		
**163**	PF13_0003 ups Rev	ACTGTATTCATGTAAAATCCATACATTCA		
**AS20**	PF13_0003 orf For	CACAGGTATGGGAAGCAATG	[3]	
**AS20**	PF13_0003 orf Rev	CCATACAGCCGTGACTGTTC	[3]	
**164**	MAL6P1.4 (PFF1580c) ups For	GTAGATATATGATAGATAGTATGGATAGAGAGAA		
**165**	MAL6P1.4 (PFF1580c) ups Rev	TTTTAGAGATAACAAAAGAGGCAAA		
**AS44**	MAL6P1.4 (PFF1580c) orf For	ATGTGTGCGAGAAGGTGAAG	[3]	
**AS44**	MAL6P1.4 (PFF1580c) orf Rev	TGCCTTCTAGGTGGCATACA	[3]	
**166**	PFL1960w ups For	CCCCATCACATATAGTACGAC TAAGAA		
**167**	PFL1960w ups Rev	TGGTAGTCACTATGTTTGTTATGATGTT		
**AS15**	PFL1960w orf For	CATCCATTACGCAGGATACG	[3]	
**AS15**	PFL1960w orf Rev	AAATAGGGTGGGCGTAACAC	[3]	
**152**	PFL0025c rif ups For	TGGTAACGTTTTATGAAAATTATGAG		
**153**	PFL0025c rif ups Rev	TGGAACCATATCTGCACTTTT		
**150**	PFL0025c rif orf For	TTGCTGTAAATGCGTGGAAAG		
**151**	PFL0025c rif orf Rev	CCGCTTCCTTAGCCGCTATAAT		
**221**	PFL2665c ups For	GATAATATAGATAGAGAGAAACAGAAGAAC		
**222**	PFL2665c ups Rev	ATTTTAGAGATAACAAAAGAGGCAAA		
**223**	PFA0005w ups For	ATAGATAGAGAGAAACGGAAGAAGATATT		
**224**	PFA0005w ups Rev	ATTTTAGAGATAACAACAGAGGCAA		
**225**	PF13_0364 ups For	AGATAGAGAGAAACAGAAGAACATTTTT		
**226**	PF13_0364 ups Rev	ATTTTAGAGATAACAACAGAGGCAAA		
**227**	PFD0615c ups For	TACATCACATCGATTACATTTTAGC		
**228**	PFD0615c ups Rev	TCTTAGTCGTATTATATGTGATGGAAA		

**229**	PF07_0049 ups For	GCGACAACCACGGCATAA		
**230**	PF07_0049 ups Rev	TGGAAAACAAACGATAAGGAAG		
**231**	PFE0005w ups For	CTACCAAACACCTACCACTCCA		
**232**	PFE0005w ups Rev	TTGTGGGTTTGCATGGTT		
**233**	PF07_0139 ups For	CAGAAGAACATATTTGCCTCTTTT		
**234**	PF07_0139 ups Rev	TTTTCCGCCTTATTTTAATTTATT		
**235**	PFD1245c ups For	GTATGGATAGAGAGAAGCAGAAGA		
**236**	PFD1245c ups Rev	TTTTAGAGATAACAAAAGAAACTATTATG		
**237**	PFD0005w ups For	CCCTTTTGTTCTCGATGTTATA		
**238**	PFD0005w ups Rev	ATATGACATATCCTGTAGTACAACGTA		
**253**	PFL0020w -800 For	GATACCCTTTTGTTCTCGATCTTAT		
**254**	PFL0020w -800 Rev	TGACATATCCTGTAGTACAACGTATATAT		
**255**	PFL0020w -600 For	TATGATAGATAATATGGATAGAGAGAAAC		
**256**	PFL0020w -600 Rev	TTTTAGAGATAACAAAAGAGGCAAA		
**257**	PFL0020w -400 For	TAAACATGTTGTATTCTTTTATATGTTTGT		
**258**	PFL0020w -400 Rev	ATGAAAAAATATTATATTTCATGTATTCCTT		
**259**	PFL0020w coding DBL5 For	AGGAACCGTTCAATCGTGACA		
**260**	PFL0020w coding DBL5 Rev	ATCCTAATGCCAACGCCACT		
MF2F	PFL2665c orf For	GCGAGGTCTTCTCGTTCTTG	[4]	
MF2R	PFL2665c orf Rev	ATGACGAAGAAGCAGCAGGT	[4]	
AS1	PFA0005w orf For	TGCGCTGATAACTCACAACA	[3]	
AS1	PFA0005w orf Rev	AGGGGTTCATCGTCATCTTC	[3]	
AS19	PF13_0364 orf For	AACCCCCAATACCATTACGA	[3]	
AS19	PF13_0364 orf Rev	TTCCCCACTCATGTAACCAA	[3]	
AS26	PFD0615c orf For	GTGCACCAAAAGAAGCTCAA	[3]	
AS26	PFD0615c orf Rev	ACAAAACTCCTCTGCCCATT	[3]	
AS30	PFD0005w orf For	GAGGCTTATGGGAAACCAGA	[3]	
AS30	PFD0005w orf Rev	AGGCAGTCTTTGGCATCTTT	[3]	
AS36	PFD1245c orf For	TCGATTATGTGCCGCAGTAT	[3]	
AS36	PFD1245c orf Rev	TTCCCGTACAATCGTATCCA	[3]	
AS38	PFE0005w orf For	TGGTGATGGTACTGCTGGAT	[3]	
AS38	PFE0005w orf Rev	TTTATTTTCGGCAGCATTTG	[3]	
AS49	PF07_0049 orf For	GACGCCTGCACTCTCAAATA	[3]	
AS49	PF07_0049 orf Rev	TTGGAGAGCACCACCATTTA	[3]	
AS53	PF07_0139 orf For	AGCAAAATCCGAAGCAGAAT	[3]	
AS53	PF07_0139 orf Rev	CCCACAGATCTTTTCCTCGT	[3]	

1. Salcedo-Amaya AM, van Driel MA, Alako BT, Trelle MB, van den Elzen AM, et al. (2009) Dynamic histone H3 epigenome marking during the intraerythrocytic cycle of Plasmodium falciparum. Proc Natl Acad Sci U S A 106: 9655-9660.
2. Duffy MF, Byrne TJ, Carret C, Ivens A, Brown GV (2009) Ectopic recombination of a malaria var gene during mitosis associated with an altered var switch rate. J Mol Biol 389: 453-469.
3. Salanti A, Staalsoe T, Lavstsen T, Jensen AT, Sowa MP, et al. (2003) Selective upregulation of a single distinctly structured var gene in chondroitin sulphate A-adhering Plasmodium falciparum involved in pregnancy-associated malaria. Mol Microbiol 49: 179-191.
4. Frank M, Dzikowski R, Amulic B, Deitsch K (2007) Variable switching rates of malaria virulence genes are associated with chromosomal position. Mol Microbiol 64: 1486-1498.
5. Dzikowski R, Frank M, Deitsch K (2006) Mutually exclusive expression of virulence genes by malaria parasites is regulated independently of antigen production. PLoS Pathog 2: e22.
